# Metrics and methods in the evaluation of prestige bias in peer review: A case study in computer systems conferences

**DOI:** 10.1371/journal.pone.0264131

**Published:** 2022-02-25

**Authors:** Eitan Frachtenberg, Kelly S. McConville

**Affiliations:** 1 Department of Computer Science, Reed College, Portland, OR, United States of America; 2 Department of Mathematics, Reed College, Portland, OR, United States of America; University of Siena, Italy, ITALY

## Abstract

The integrity of peer review is essential for modern science. Numerous studies have therefore focused on identifying, quantifying, and mitigating biases in peer review. One of these better-known biases is prestige bias, where the recognition of a famous author or affiliation leads reviewers to subconsciously treat their submissions preferentially. A common mitigation approach for prestige bias is double-blind reviewing, where the identify of authors is hidden from reviewers. However, studies on the effectivness of this mitigation are mixed and are rarely directly comparable to each other, leading to difficulty in generalization of their results. In this paper, we explore the design space for such studies in an attempt to reach common ground. Using an observational approach with a large dataset of peer-reviewed papers in computer systems, we systematically evaluate the effects of different prestige metrics, aggregation methods, control variables, and outlier treatments. We show that depending on these choices, the data can lead to contradictory conclusions with high statistical significance. For example, authors with higher h-index often preferred to publish in competitive conferences which are also typically double-blind, whereas authors with higher paper counts often preferred the single-blind conferences. The main practical implication of our analyses is that a narrow evaluation may lead to unreliable results. A thorough evaluation of prestige bias requires a careful inventory of assumptions, metrics, and methodology, often requiring a more detailed sensitivity analysis than is normally undertaken. Importantly, two of the most commonly used metrics for prestige evaluation, past publication count and h-index, are not independent from the choice of publishing venue, which must be accounted for when comparing authors prestige across conferences.

## Introduction

Scientists usually publish their research findings in peer-reviewed venues such as journals and conferences. Their work is judged by a small set of peers from their field, who typically remain anonymous so that their critique can remain free from author influence. This so-called single-blind (SB) reviewing process has been widely accepted as the baseline standard of credibility of publications in the scientific process [1]. However, single-blind reviewing still lets reviewers know the identities of the work’s authors. Several researchers have argued that this knowledge can affect the review outcomes in subjective ways. In particular, when reviewers encounter a famous author or affiliation they may subconsciously experience authority bias, where higher accuracy is attributed to the opinion of authority figures [2]. This bias is also known as “the halo effect,” “status bias,” or “prestige bias,” the term we use in this paper. It has been implicated as an important but unscientific factor in the review process, which in turn can lead to bad science, lower credibility for the scientific process as a whole, and an exacerbation of the underrepresentation of minorities in research [3].

A commonly suggested antidote to prestige bias is double-blind (DB) reviewing, where authors’ identities and affiliations are hidden from reviewers. Not all publication venues employ DB reviewing, both for practical and principled considerations. However, the coexistence of SB and DB reviewing (sometimes in the same venue) opens the door to comparative studies of the effects of prestige bias. For example, the association between reviewing policy and various biases has been assessed in disciplines as disparate as economics [4], behavioral ecology [5, 6], ophthalmology [7], medicine [8, 9], psychology [10], and computer science [3, 11]. Some of these studies have found conclusive evidence for bias while others have found none, sometimes even in the same subfield.

Often, these studies are not directly comparable because of various differences in the data, methodologies, or metrics used. One limitation of these studies is that there are various ways to estimate a person’s name recognition, and they do not all yield the same results. Another limitation is that the reviewing policy of a venue is not usually independent from other factors, so DB reviewing could be confounded with other factors that affect submission or acceptance decisions. When we tried to apply similar methodologies to one broad field of research, computer systems, we discovered that the outcome can vary significantly based on the methodology and metrics used.

The present work began as one of these studies, focusing on the field of computer systems. We soon realized that our particular dataset was insufficient to provide conclusive evidence for or against prestige bias. We also realized that the limitations we encountered may actually be shared by many of these studies and had not yet been systematically compared and documented. The focus of our study thus became the exploration of how subtle changes in the data and methods used to detect prestige bias can radically change the resulting outcome.

The primary contribution of this paper is therefore an analysis of the sensitivity of the association between reviewing policy and prestige bias. To reiterate, this study does not attempt to resolve the question of the existence of prestige bias in this field. Instead, we focus on exposing and analyzing the factors that could change the outcome of studies that do attempt to address this question. These factors include questions such as: “how to estimate the prestige or reputation of authors?” “where should data be sourced from, and how should it be cleaned?” and “how should we correct for confounding variables like conference prestige?” A meaningful analysis of review bias requires a careful consideration of these factors, and a cognizant effort to address the sensitivity of the outcomes to these choices. Understanding these factors can help contribute to more reliable and stable results in future studies on bias in general, and prestige bias in particular.

The rest of this paper is organized as follows. We start by describing the extensive dataset we collected on our field of focus. The next section then describes empirical evidence for and against prestige bias, based on the types of metrics, their aggregations, statistical analyses performed, and data cleaning choices. This section demonstrates how contradictory but statistically significant results can be obtained by different approaches, despite working on the very same dataset. We elaborate and discuss these findings and practical recommendations in the Discussion. Finally, we review related work and provide some conclusions and recommendations.

## Materials and methods

The primary dataset we analyze comes from a hand-curated collection of 56 peer-reviewed systems and systems-related conferences from a single publication year (2017). In computer science, and in particular in its more applied fields such as systems, original scientific results are typically first published in peer-reviewed conferences [12–15], and then possibly in archival journals, sometimes years later [16]. The conferences we selected include some of the most prestigious systems conferences (based on indirect measurements such as Google Scholar’s metrics), as well as several smaller or less-competitive conferences for contrast (Table 1, with additional details in the S1 Appendix). We chose to focus on a large cross-sectional set of conferences from a single publication year (2017), to reduce variations in time. Our choice of which conferences belong to “systems” is necessarily subjective. For the purpose of this study, we define systems as the study and engineering of concrete computing systems, which includes research topics such as: operating systems, computer architectures, data storage and management, compilers, parallel and distributed computing, and computer networks. Obviously, not all systems papers from 2017 are included in our set, and some papers that are in our set may not necessarily be considered part of systems (for example, if they lean more towards algorithms or theory). However, we believe that our cross-sectional set is both wide enough to represent the field well and focused enough to distinguish it from the rest of computer science. In total, our sample includes 2439 accepted peer-reviewed papers.

**Table 1:** System conferences and review policy with four reputation or prestige metrics: Acceptance rate, total number of paper submissions in 2017, age of conference in years, and mean historical citations per paper, which are all correlated (*p* < 0.01). Conferences are sorted by descending acceptance rate.

Conference	Double-blind	Acceptance rate	Submissions	Age	Citations/paper
KDD	No	0.09	747	19	16.9
ICDM	Yes	0.09	778	17	7.9
SIGMETRICS	Yes	0.13	203	44	11.8
SP	Yes	0.14	419	38	24.4
SIGCOMM	Yes	0.14	250	40	19.6
PLDI	Yes	0.15	322	38	33
IMC	No	0.16	179	16	24.4
NDSS	Yes	0.16	423	20	Unknown
NSDI	Yes	0.16	254	13	40.7
ISCA	Yes	0.17	322	44	23.3
SOSP	Yes	0.17	232	50	45.9
ASPLOS	Yes	0.18	320	35	35.5
CCS	Yes	0.18	836	24	14.3
MICRO	Yes	0.19	327	50	17.3
SC	Yes	0.19	327	29	7.6
CoNEXT	No	0.19	171	13	6.2
MobiCom	Yes	0.19	186	23	18.8
HPDC	No	0.19	100	25	7.1
ICAC	No	0.19	73	14	Unknown
SIGMOD	Yes	0.2	489	47	24.4
SIGIR	No	0.22	362	46	17
ATC	No	0.22	277	25	Unknown
EuroSys	Yes	0.22	188	11	13.7
PPoPP	Yes	0.22	132	29	12.8
HPCA	No	0.22	224	22	18.1
HiPC	No	0.22	184	24	2
IPDPS	No	0.23	508	31	5.1
PACT	Yes	0.23	108	25	9.5
FAST	Yes	0.23	116	15	15.8
MASCOTS	No	0.24	84	25	4.5
SPAA	No	0.24	127	29	9.2
PODC	No	0.25	154	35	11.6
CCGrid	No	0.25	286	16	6.3
Middleware	Yes	0.26	77	18	6.2
CLOUD	No	0.26	110	10	4.9
EuroPar	No	0.28	176	23	Unknown
ICPP	No	0.29	210	46	Unknown
PODS	No	0.29	101	36	27
ISPASS	Yes	0.3	81	17	8.8
OOPSLA	Yes	0.3	223	31	9
Cluster	No	0.3	217	18	4.2
HotOS	No	0.31	94	30	Unknown
HotCloud	No	0.33	58	9	Unknown
ISC	Yes	0.33	66	31	Unknown
HotI	No	0.33	39	25	Unknown
SYSTOR	No	0.34	47	10	3.4
ICPE	No	0.35	83	19	4.9
HotStorage	No	0.36	58	9	Unknown
IISWC	Yes	0.37	83	19	11.8
CIDR	No	0.41	78	8	Unknown
VEE	Yes	0.42	43	13	13
SLE	No	0.42	57	10	Unknown
HPCC	No	0.44	176	19	Unknown
HCW	No	0.47	15	26	Unknown
SOCC	No	Unknown	Unknown	8	18.6
IGSC	No	Unknown	Unknown	8	Unknown

To resolve decisively the question of prestige bias in computer systems, we would need to compare the prestige of accepted authors to that of rejected authors, for which no public information is available. So instead of resolving the question of the existence of prestige bias, we concentrate on a related question: Is there a statistically significant difference in the rates of famous authors published in SB or DB conferences?

To answer this question, we collected extensive information to help us estimate the prestige of authors and conferences. For each conference, we downloaded all papers and gathered information about all authors, program committee (PC) members, and other roles. Conferences do not generally offer information on authors’ demographics, but we were able to unambiguously link approximately two thirds (65.7%) of researchers in our dataset to a Google Scholar (GS) profile [17]. For each author and PC member, we collected all metrics in their GS profile, such as total previous publications (ca. 2017), h-index, etc. Note that we found no GS profile for about a third of the researchers (36.06%), and these researchers appear to be less experienced than researchers with a GS profile. We therefore also considered a proxy metric for author experience (total number of past publications) from another source, the Semantic Scholar (S2) database [18], which is how we discovered that most missing GS profiles represent inexperienced authors.

In addition to researcher information, we gathered various statistics on each conference, either from its web page, proceedings, or directly from its chairs. These data included review policies, the composition of its technical PC, and the number of submitted papers. On these conferences, we also collected historical metrics from the Institute of Electrical and Electronics Engineers (IEEE), Association for Computing Machinery (ACM), and Google Scholar websites, including past citations, age, and total publications.

### Limitations

Perhaps the most significant limitation of this study (and many similar studies) is a noisy dataset. In fact, addressing missing and erroneous data is one of the major focal points of this study. We collected most reputation metrics from the Google Scholar database because it includes many metrics and allows for manual verification of the identity of each author by linking to their homepage. This database is not without its limitations, however. It does not always disambiguate author names correctly, and it tends to overcount publications and citations [19–22].

We have attempted to mitigate these limitations as follows:

- For the number of past publications metric, we also include data from the Semantic Scholar (S2) database, which has near-perfect coverage. This metric is strongly correlated across these two databases, suggesting that authors that are “famous” according to GS are likely also independently “famous” based on the S2 database. Since we only care about relative “fame”, GS’s exaggerated metric values do not affect our analysis. Indeed the choice of database did not materially change our results for or against prestige bias in each case.
- We addressed the name disambiguation challenge by manually verifying the GS profiles of all researchers and ensuring that they include the papers from our dataset and do not mix together multiple researchers from different fields. Ambiguous profiles were omitted from our dataset.
- Virtually all databases are expected to have some missing data, which is a methodological concern we address in the paper in the section on data imputation. Specifically, because most missing GS profiles were of relatively inexperienced researchers (based on S2 metrics), we were able to impute or extrapolate experience metrics for all missing profiles and show that they do not significantly change the evaluation of prestige bias in our dataset.

An additional limitation of our methodology is that it is constrained by the manual collection of data. We chose to collect each conference and author statistics manually to control its quality and alleviate some of the concerns around name disambiguation. However, the effort involved in compiling all the necessary data limits the scalability of our approach to additional conferences or years. In particular, limiting our dataset to a single publication year means that it cannot be used to evaluate prestige bias over time, for example as authors’ names grow more recognizable with experience. But it does eliminate time-based variance within and across conferences.

### Statistics and source code

For statistical testing, group means were compared pairwise using Welch’s two-sample t-test and group medians using the Wilcoxon Signed Rank Test; differences between distributions of two categorical variables were tested with the *χ*^2^ test; and comparisons between two numerical variables were evaluated with Pearson’s product moment correlation coefficient. In terms of statistical modeling, fixed-effects and mixed-effects generalized linear models were built and Satterthwaite’s degrees of freedom method is used for hypothesis testing on model coefficients. All statistical tests are reported with their p-values.

All analyses were conducted in the statistical software package R [23]. The specific code for this study can be found in the file pubs/prestige/prestige.Rmd and the relevant R packages in the file pubs/dependencies.R. A Docker image with the complete software environment for reproduction of this analysis can be found at https://hub.docker.com/repository/docker/eitanf/sysconf under the prestige tag.

### Ethics statement

The data collected for this study was sourced from public-use datasets such as conference and academic web pages. This study was exempted from the informed consent requirement under Exempt Category 4: the use of secondary data by Reed College’s Institutional Review Board (No. 2021-S26).

## Empirical results

In this section, we explore the observed relationships between reputation and reviewing policy across four design dimensions, or factors: metrics of reputation, aggregation of prestige across coauthors, additional explanatory (confounding) variables, and approaches to data cleaning.

### Reputation metrics

**Fig 1:**
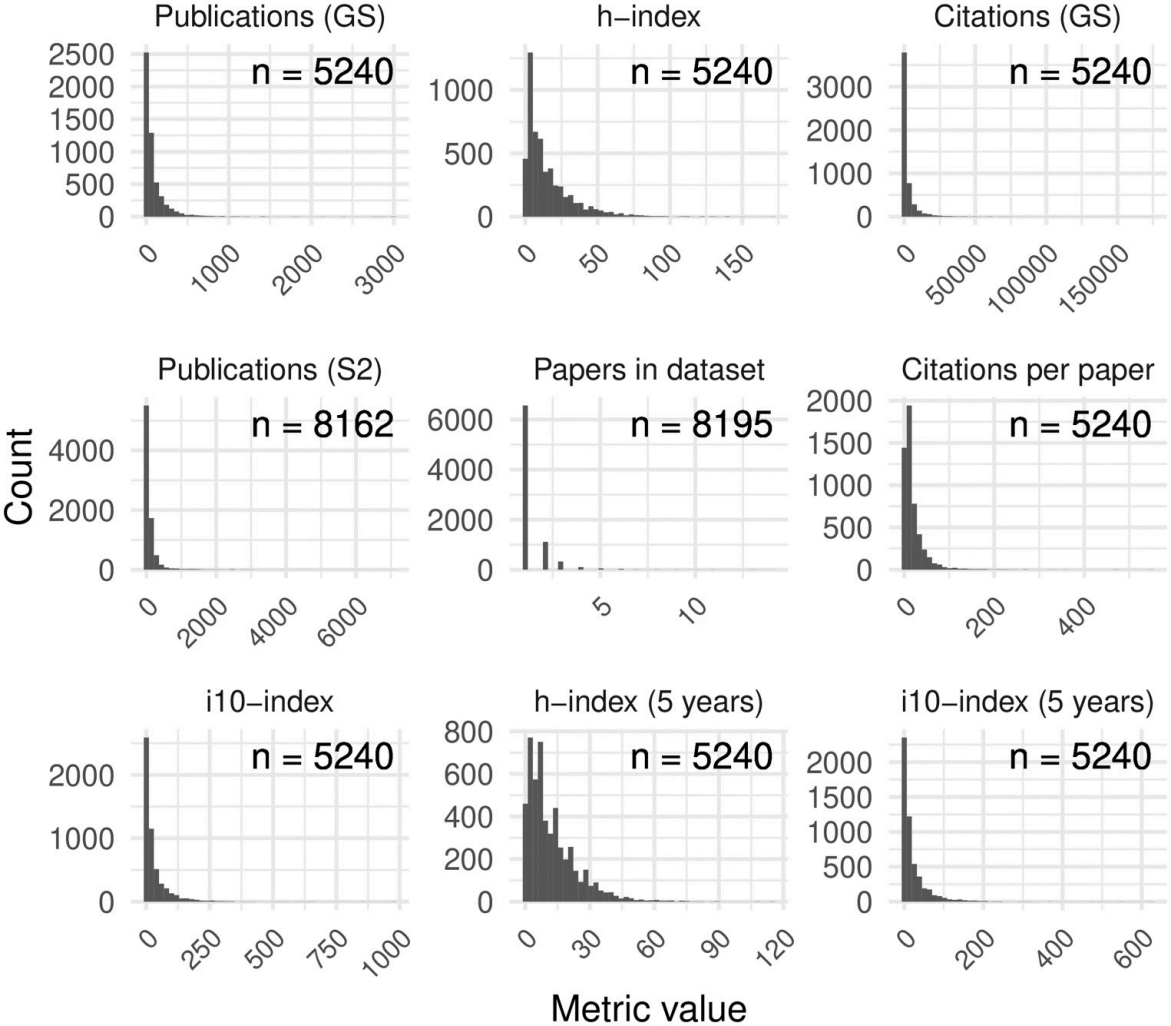
Histograms of the author-level prestige proxies. The number of non-missing values (n) is provided for each variable.

Author reputation is an intangible, abstract concept. Nevertheless, the quantitative methodology that we focus on requires a concrete approximation. As a proxy for reputation, most bibliometric studies employ metrics based on productivity (publication counts), impact (citation metrics), or both [24, 25]. We chose an initial list of nine such metrics to analyze, whose distributions are depicted in Fig 1.

This list is certainly not exhaustive; the debate on their relative merits and effectiveness is ongoing, and new and improved metrics are proposed regularly. But this list represents some of the most commonly used metrics, and as our results show, is sufficient to surface diverse and even contradictory results in the context of reviewing policy. We next describe these metrics and their characteristics in our dataset.

#### Publication counts

Counting the number of past publications is ostensibly the most straightforward way to quantify how well-known authors are. After all, the more papers that carry their name, the more likely it is that their name is recognized by reviewers. But the relationship is hardly linear in practice:

- Not all papers are equally well-known, read, or cited—a problem that the impact-based metrics attempt to address.
- Rising researchers may be receiving significant name recognition without having yet accumulated a long publication record.
- The decision of what counts as a “publication” can be subjective and vary from database to database. For example, do patents count? preprints? blog posts?
- Accurate collection of even the raw data is complicated by factors such as author name disambiguation, paper disambiguation, and noisy or crowd-sourced data.
- Systems papers in particular, like in other experimental fields, vary significantly in number of coauthors because some implementations take a large team effort. Credit attribution therefore becomes particularly tricky in this field, also when using the h-index [26].

With these limitations in mind, we start our analysis by looking at the count of previous publications for each author. For each accepted paper, we looked at the publication count of the most prolific coauthor to capture the potential name recognition for the paper as a whole. In our dataset, authors in SB conferences average a higher GS publication count than in DB conferences (mean SB: 272.44, DB: 230.83; *t* = 3.202, *p* < 0.01). This relationship appears to support a hypothesis of prestige bias for these conferences (to reiterate, there is insufficient evidence for a conclusive causal relationship). When we compare the publication counts from our other data source, Semantic Scholar, which covers all authors, we find similar evidence, albeit weaker: (mean SB: 332.25, DB: 305.49; *t* = 1.483, *p* = 0.14).

We can also use a third source of paper counts to differentiate between active and mostly inactive researchers, using only current statistics. If we look at the number of papers published in our cross-section of conferences from 2017, we find that SB conference authors actually average significantly *fewer* publications than those in DB conferences (mean SB: 2.85, DB: 3.29; *t* = −4.468, *p* < 10^−5^). This result could provide evidence against a prestige bias, but it could also point to a completely different hypothesis, such as authors preferring to submit to DB conferences for that specific point in time. The evidence based on publication counts alone is inconclusive.

#### Citation-based metrics

We next turn our attention to another set of commonly used metrics, those incorporating citation count, which is how academic impact is typically measured. Citation-based metrics try to capture the fact that not all publications have the same influence on other researchers, and indirectly, on the author’s reputation. Note that the correlation between total past citations and total past papers is not particularly high, leading potentially to divergent results when evaluating prestige bias (Pearson correlation of *r* = 0.56 when using GS paper counts; *r* = 0.29 with S2 data).

**Fig 2:**
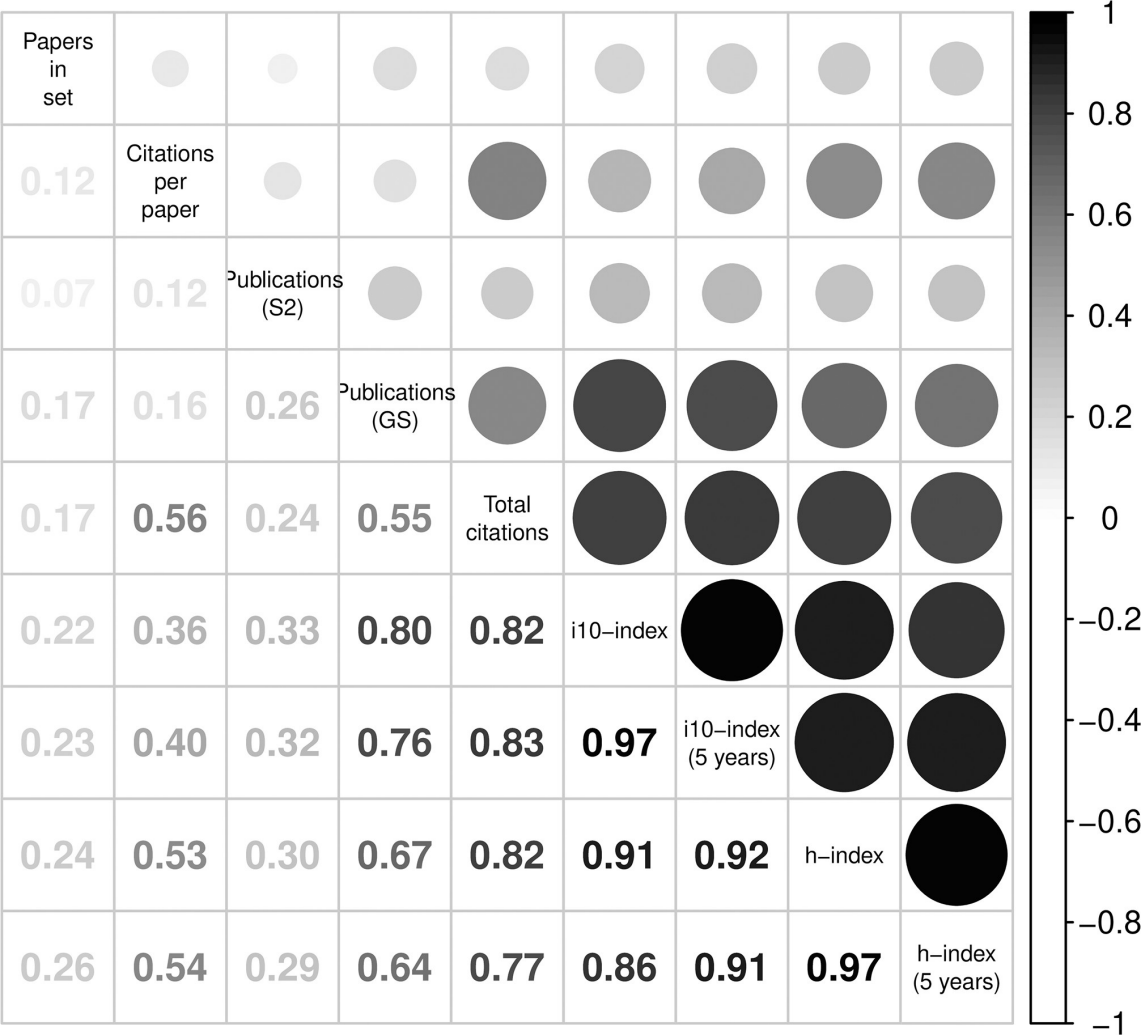
Correlation matrix for the author-level prestige metrics.

From GS, we collected for each author with an identifiable profile their total number of past citations, h-index, i10-index (number of papers with 10 or more citations), the h-index over the past 5 years and the i10-index over the past 5 years. We omit the last two metrics from our discussion because of their high correlation with h-index and i10-index, respectively (Fig 2). We also computed another metric from these statistics, citations per paper, by dividing total GS citations with total GS publications.

Once more, we look at the most experienced coauthor in each paper group, and compare the averages across SB and DB papers. Of these, the citations-per-paper metric shows the strongest association with DB reviewing (mean SB: 34.23, DB: 45.5; *t* = −6.593, *p* < 10^−9^). The h-index metric also strongly indicated higher values in DB conferences (mean SB: 33.39, DB: 35.83; *t* = −2.593, *p* < 0.01).

The total citations metric shows a much weaker, nonsignificant association in the same direction (mean SB: 8377.72, DB: 8908.46; *t* = −0.886, *p* = 0.38), while the i10 metric shows a nonsignificant association in the opposite direction (mean SB: 90.14, DB: 85.32; *t* = 1.231, *p* = 0.22). Again, the evidence for or against prestige bias is inconclusive.

#### Program committee metrics

Two different reputation metrics that are not regularly reported or measured reflect overall participation of the authors in review roles. Many studies on prestige bias focus on journal authors, where most reviewers typically remain unidentified. But in computer science, and systems in particular, where the main publication venue is conferences, most of the peer review is carried out by broad, publicized program committees (PCs). A researcher’s participation in these PCs is related to their reputation in two ways, as follows.

First, to be invited to serve in a PC, a researcher is usually regarded as an authority in the field. We can extend this intuition to reputation metrics, either as a quantitative one—how many PCs in our set an author belongs to—or a qualitative one, simply noting whether they participate in any PC at all. For the former, we observe that the coauthors with the highest PC participation in each paper group average marginally more PC roles in DB conferences (mean SB: 1.25, DB: 1.29; *t* = −0.797, *p* = 0.43). For the latter, we observe a null result: the ratio of papers authored by at least one member of a PC in our set is actually lower for DB conferences than for SB (63.2% vs. 60.1%; *χ*^2^ = 2.32, *p* = 0.13).

Second, when a person submits a paper to a specific SB conference wherein they also serve on the PC, they are quite likely recognized by reviewers, who also compose the PC. We can compute the percentage of papers in each conference where at least one of the authors participates in the PC for that same conference. Comparing across review polices, we find no significant difference in the mean percentage for DB and SB conferences (25% vs. 27%; *t* = −0.38, *p* = 0.71).

In summary, neither PC metric for author reputation shows a strong association with the review policy of the conference.

### Paper aggregations

The analysis thus far made two implicit assumptions on the likelihood that a paper would be recognized as “reputable”: that the author with the highest reputation metric determines the reputation of the paper, and that the likelihood increases linearly with the value of the reputation metric. In this section, we relax these assumptions. We first look at the effect of summarizing a paper’s author reputation based on statistics other than maximum value. Then, we look at qualitative (categorical) classifications of a paper’s potential for recognition.

#### Aggregations by paper

The typical systems paper has a large number of coauthors compared to other disciplines, perhaps because of its emphasis on concrete systems that can require significant team effort to implement. In our dataset, papers average 4.42 authors per paper (SD: 2.63). We have assumed so far that a paper is most likely to be recognized based solely on the author with the highest reputation metric. But other authors may play an important role in name recognition, as well as the whole group.

For example, the total reputation of the author group could also play a potential role in the reviewer’s bias. It could therefore be interesting to compare total paper reputation to review policy (omitting all papers that had one or more missing reputation values, where a partial sum is meaningless). Alternatively, we can look at the mean or median reputation (omitting only missing coauthors, not entire papers). Finally, we can aggregate papers by looking only at the reputation of the first or last author. Systems papers usually list the primary contributor in the first author position, while the head of the lab is often listed last; in our dataset, only 12% of papers with three or more coauthors ordered the author list alphabetically.

**Fig 3:**
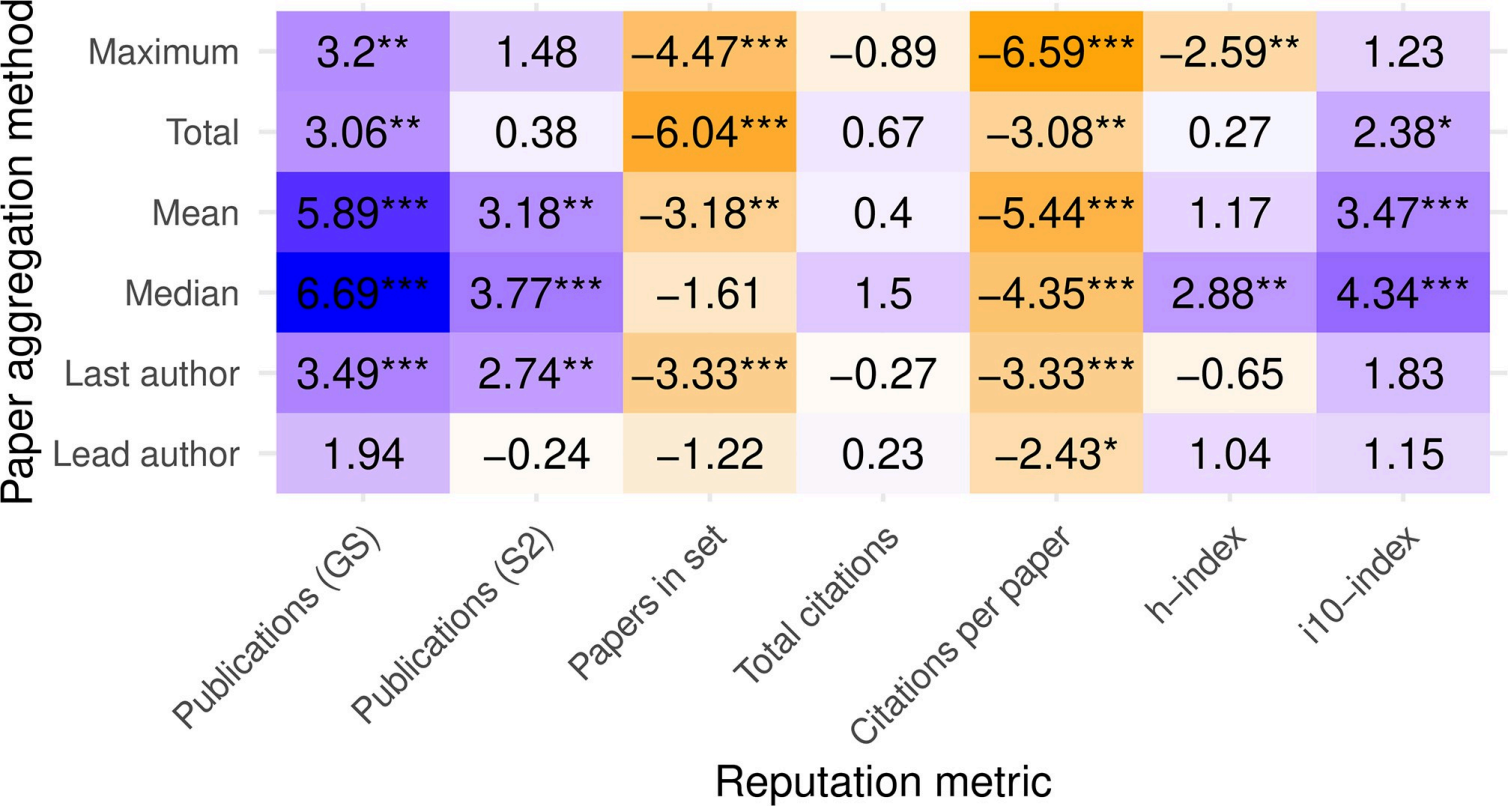
Test statistics for difference in means with various reputation metrics and aggregations across papers using two-sided Student’s t-test. Positive t-statistic values (purple) indicate higher reputation in SB conferences. Darker colors reflect larger-magnitude values, normalized by columns. Negative values (orange) indicate higher reputation in DB conferences. Stars indicate p values below 0.05, 0.01, and 0.001, respectively.

Looking at all six aggregation methods across seven reputation metrics reveals a mixed picture (Fig 3). The first row—using maximum reputation per paper aggregation—repeats the results we discussed in the previous section. All other rows represent new aggregations. Depending on one’s choice of metrics and aggregations, one can measure statistically significant reputation bias, anti-bias, or nonsignificant bias. Any statistically sound evidence to suggest bias can find a counter-argument just as sound in the same dataset.

Nevertheless, we can discern a few consistent properties from these comparisons:

- The citations-per-paper and papers-in-set metrics indicate lower mean reputation in SB conferences across all paper aggregations. It is noteworthy because these two metrics are completely disjoint in time: one looks at the past, and one at the present.
- Conversely, measuring reputation by number of publications results in higher mean reputation for SB conferences, regardless of aggregation or database used.
- However, using the more complete S2 database lowers the t-statistic values and significance. This result may be unsurprising, as it incorporates more data on the lower side of the reputation distribution; but it serves as a cautionary tale for ascribing statistical significance from incomplete databases.
- Similarly, aggregating papers by the reputation of the lead author results in the least conclusive findings. We believe that many lead authors are relatively inexperienced students.
- Looking at each paper’s median reputation metric instead of mean leads to higher t-statistic values, i.e., higher indicated reputation in SB conferences and higher likelihood of prestige bias. The p-values are also lower, likely because the standard error of the median is smaller than that of the mean since it is less susceptible to outliers.

Related to the last point, we can look not only at median aggregation per paper, but also across papers. That is, we can compare how the median reputation per paper (however it is aggregated) differs between SB and DB conferences, again with the purpose of attenuating the effect of large right-skewed outliers.

**Fig 4:**
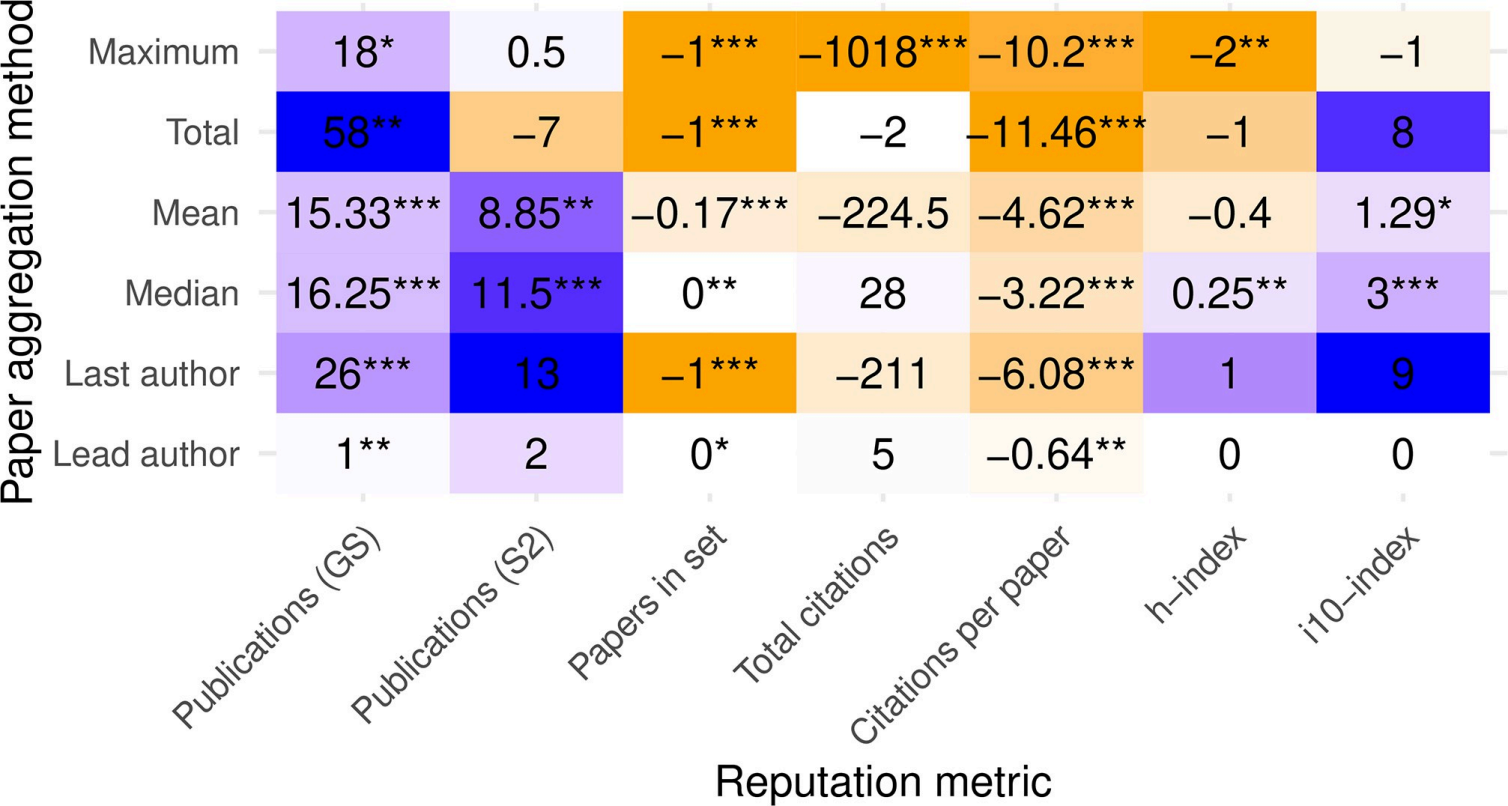
Difference in medians for various reputation metrics and aggregations across papers using two-sided Wilcoxon test. Positive values (purple) indicate higher reputation in SB conferences. Negative values (orange) indicate higher reputation in DB conferences. Darker colors reflect larger-magnitude values, normalized by columns. Stars indicate p values (from a Wilcoxon two-sided unpaired test) below 0.05, 0.01, and 0.001, respectively.

Comparing Figs 3 and 4 shows that many of the metrics and paper aggregations agree in direction (sign) whether looking at the mean values across SB/DB or medians, although confidence levels vary greatly between the two. Observe for example how the total-citations metric went from nonsignificant means to highly significant medians, perhaps because this metric’s values are the most widely distributed and right-skewed, thereby creating large differences between means and medians.

The implication from this comparison is, again, that there exist combinations of metrics and aggregations, all arguably representing reasonable choices, that can change the outcome from statistical significance to nonsignificance. As additional confirmation, a 2006 study found no significant difference in the mean publications of authors in two conferences [27], but a followup study on the same dataset found evidence for prestige bias when comparing across medians [28]. The choices for metrics and aggregations must therefore be carefully justified, compared, and balanced to increase the credibility of such analyses.

#### Binary classifications of fame

Up to this point, we have treated the reputation or fame of researchers as a continuous variable. This treatment allowed us to answer the question of whether the likelihood of an author to publish in SB proceedings increases commensurate with their reputation metrics. But the question of reputation bias may instead be more binary, based on the accept/reject outcome of a paper: either a paper is accepted based on an author’s reputation being recognized or it is not. This qualitative classification has the advantage of avoiding the problem of the long tails of the reputation metrics (see Fig 1) that could skew the mean reputation of a sample.

The quantitative question then becomes, what is the proportion of accepted papers in SB and DB conferences for which at least one of the authors carries enough reputation to be recognized? Do these proportions vary significantly by the conference reviewing policy? Since we do not know a-priori what “enough reputation” means, we can measure how these proportions change based on different reputation metrics and thresholds.

**Fig 5:**
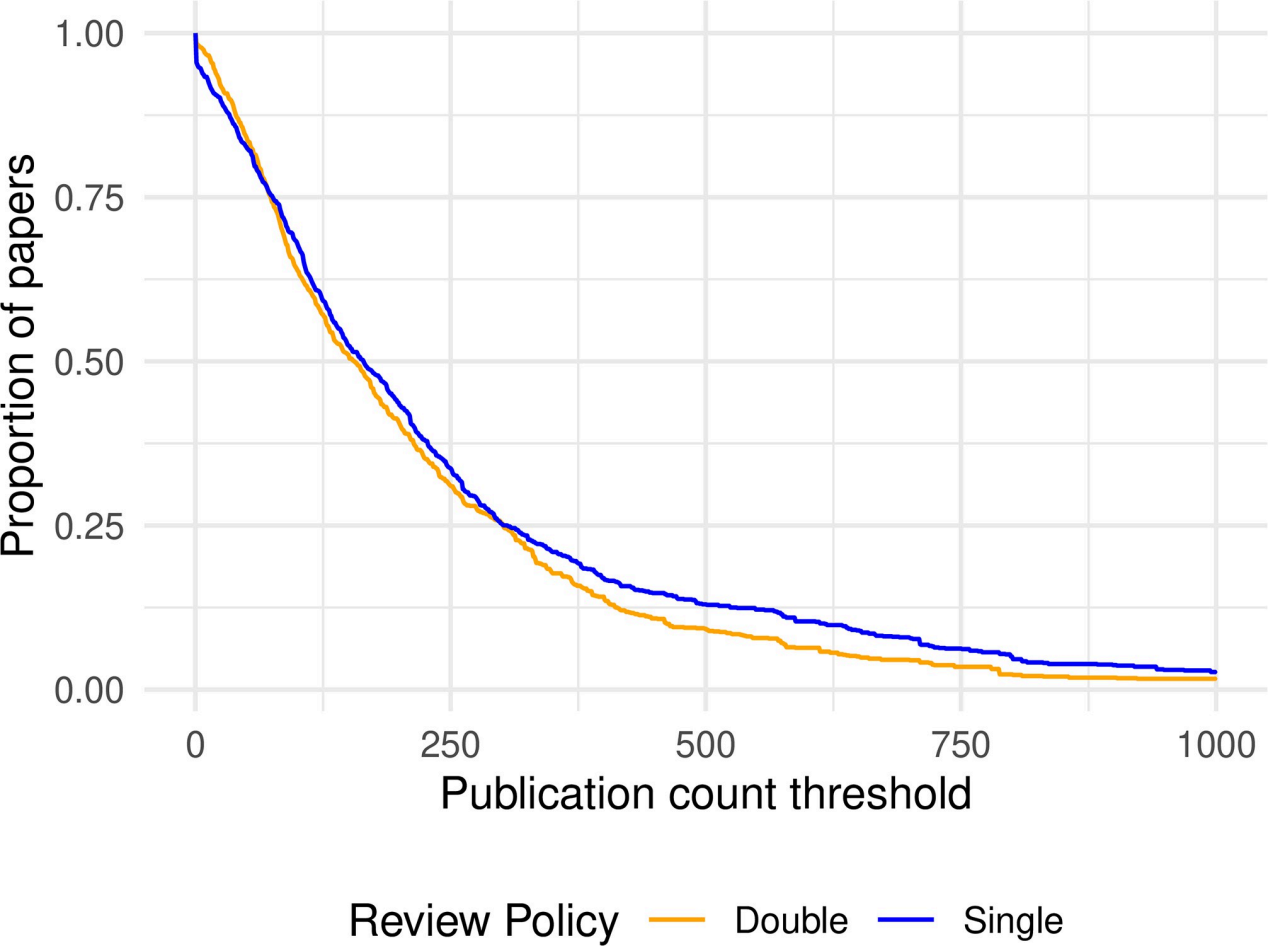
Proportion of papers that have at least one author with at least as many publications as the threshold (Google Scholar data).

For example, Fig 5 shows the proportion of papers written by famous authors in SB or DB conferences, based on number of publications. It shows that beyond a certain level of reputation (around 100 papers), famous authors are always overrepresented in SB conferences, consistent with a possible prestige bias. Comparing these proportions across SB and DB conferences, the *χ*^2^-test-derived p-values are below 0.05 for 75.7% of the threshold values.

**Fig 6:**
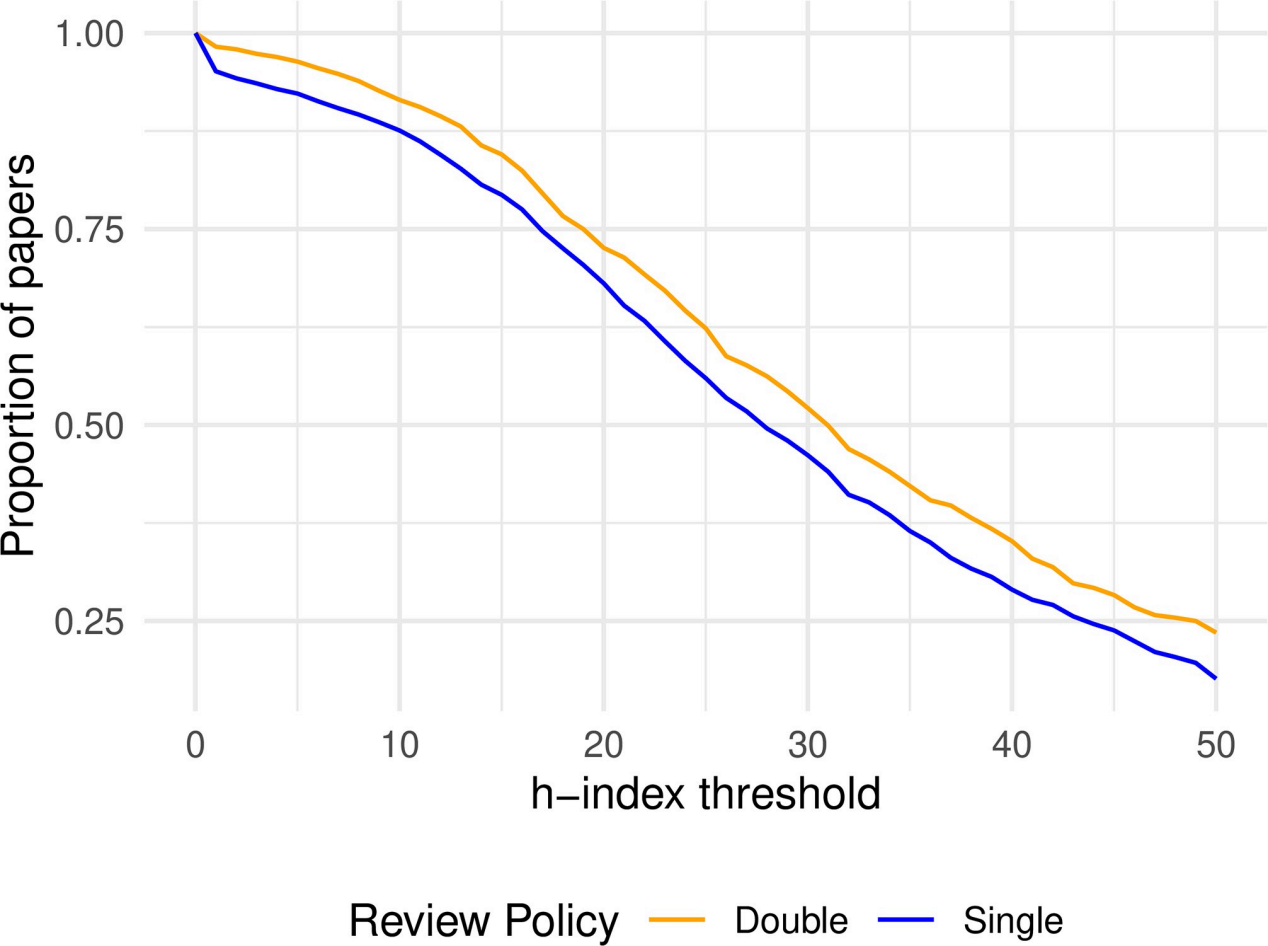
Proportion of papers that have at least one author with an h-index value above the threshold.

The two lines are even more distinctly divergent when using h-index for reputation, but in reverse. Fig 6 shows that papers by famous authors in DB conferences consistently outnumber those in SB conferences by a near-constant amount of some 5 to 10 percentage points. Again, p-values below 0.05 show up for 72% of the threshold values.

These two examples show strong statistical evidence with remarkable insensitivity to the threshold chosen, but in opposite directions. They demonstrate once more how critically the conclusion for or against prestige bias can depend on the chosen reputation metric.

#### Banded classification of experience

Another related approach to categorize experience was described in the original h-index study [29]. It proposed that researchers may fall into experience bands, roughly corresponding to novice/assistant professor, mid-career/associate professor, and experienced/full professor. Based on his observations in the field of physics, Hirsch proposed h-index limits of under 11, between 12 and 17, and 18+ to distinguish among these bands.

**Fig 7:**
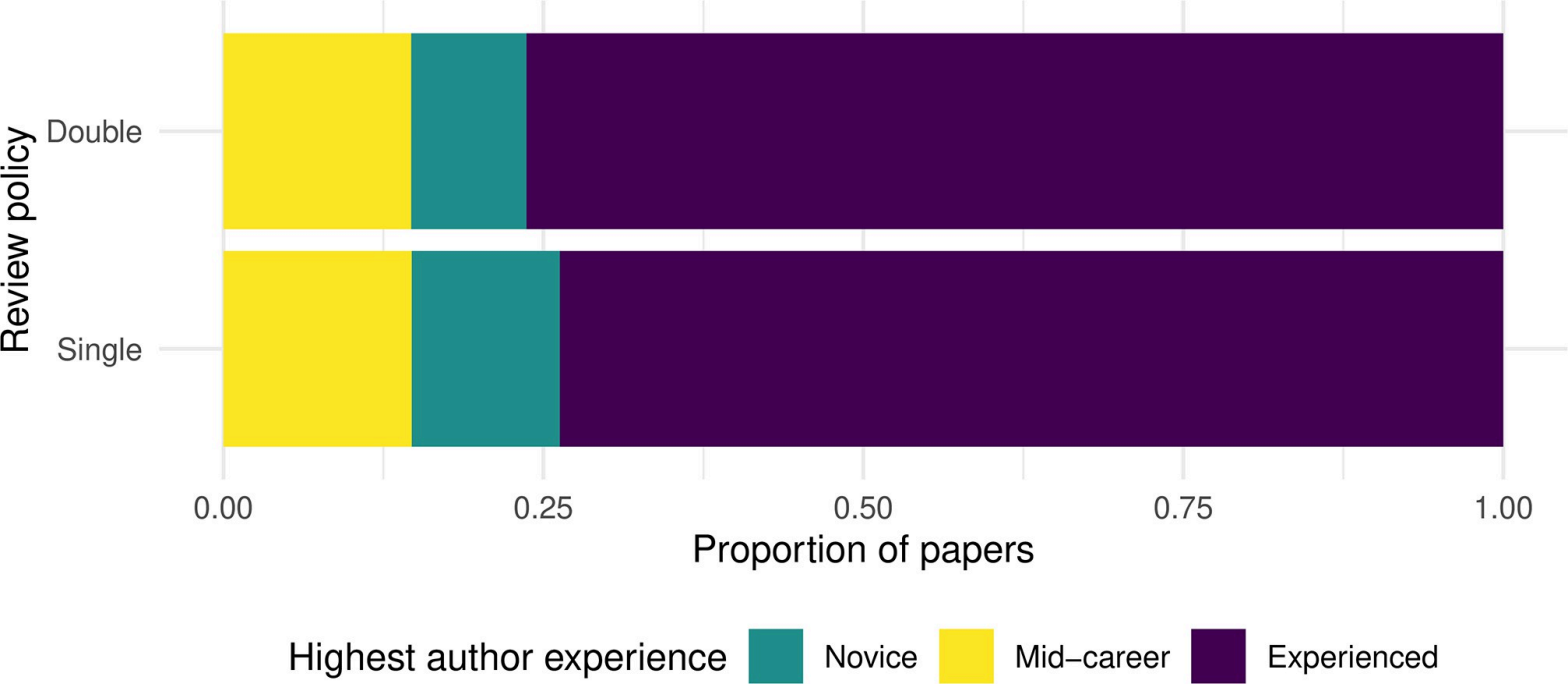
Experience bands of papers’ authors by review policy. Each paper is counted exactly once, based on the highest h-index among its coauthors.

When we apply these (admittedly arbitrary) limits to our dataset (Fig 7), we do not find a statistically significant difference in the distribution of senior author experience between the reviewing policies (*χ*^2^ = 4.27, *p* = 0.12). This null result is consistent with the evidence we uncovered in previous sections using maximum h-index, both for quantitative and qualitative aggregations.

#### Prestige by affiliation

To complete our exploration of paper aggregations, we look at one more common method to approximate name recognition of authors, based on their affiliation. For this analysis, we follow the methodology of Tomkins, Zhang, and Heavlin from their study examining prestige bias in the 2017 Web Search and Data Mining (WSDM) conference [3]. Their study tagged a paper as coming from a “top university” if any of its coauthors is affiliated with one of the top 50 universities per www.topuniversities.com (27% of papers). They similarly tagged a paper as coming from a top company if any author was affiliated with either Google, Microsoft, Yahoo!, or Facebook (18% of papers).

Since our data comes from the same publication year as theirs, we used exactly the same criteria for tagging for an apples-to-apples comparison. Unfortunately, the original study and supporting information does not contain the list of universities or how it was processed. We therefore downloaded the list of the top 50 “Engineering and Technology” universities and saved it as a list of internet domain names (see S1 Dataset).

In our dataset, 31.9% of papers had at least one coauthor from a top university, and 7.1% of papers had at least one coauthor from a top company. The percentage of papers for authors from a top university in DB conferences is significantly higher than in SB conferences (35.5% vs. 28.3%, *χ*^2^ = 14.4, *p* < 10^−3^) The same can be said for top-company affiliation (9% vs. 5.3%, *χ*^2^ = 12.33, *p* < 10^−3^).

It appears that this aggregation, like total citations or maximum h-index, but contrary to Tomkins’ results, is consistent with anti-prestige bias (higher prestige in DB conferences). Unlike Tomkins’ results, however, our data does not come from a controlled experiment on a single conference, so it cannot provide counterfactual evidence.

### Confounding variables

The next assumptions we dismantle are that the reviewing policy of a conference is an independent, random variable and that each paper is a independent observation. In this section, we ask the question, how would our findings on prestige bias change if we examined them in a larger context of conference factors, and after accounting for conference-level dependencies in the data?

A conference choice of review policy is often connected to other factors that may also be associated with the experience of researchers submitting to it. Specifically, DB conferences tend to have a **longer history** in years (*p* = 0.02), a **lower acceptance rate** (*p* < 0.01), **higher H5-index** in GS (*p* < 0.01), **more pages per paper** (*p* < 10^−4^), **more paper submissions** (*p* = 0.05), with **more coauthors per paper** (*p* < 0.01), a **lower ratio of authors coming from the PC itself** (*p* < 0.01), and more **citations to past papers** (*p* = 0.14), especially when normalized to **citations per paper** (*p* = 0.01).

In other words, DB conferences themselves show several distinct characteristics, some of which suggest higher reputation. The higher implied prestige of the conference could be associated with the prestige of authors as well, confounding the relationship. Additionally, papers published in a particular conference are likely more correlated with each other than papers published across conferences. Therefore, we should account for this correlation structure in our models directly.

#### Linear regression

**Fig 8:**
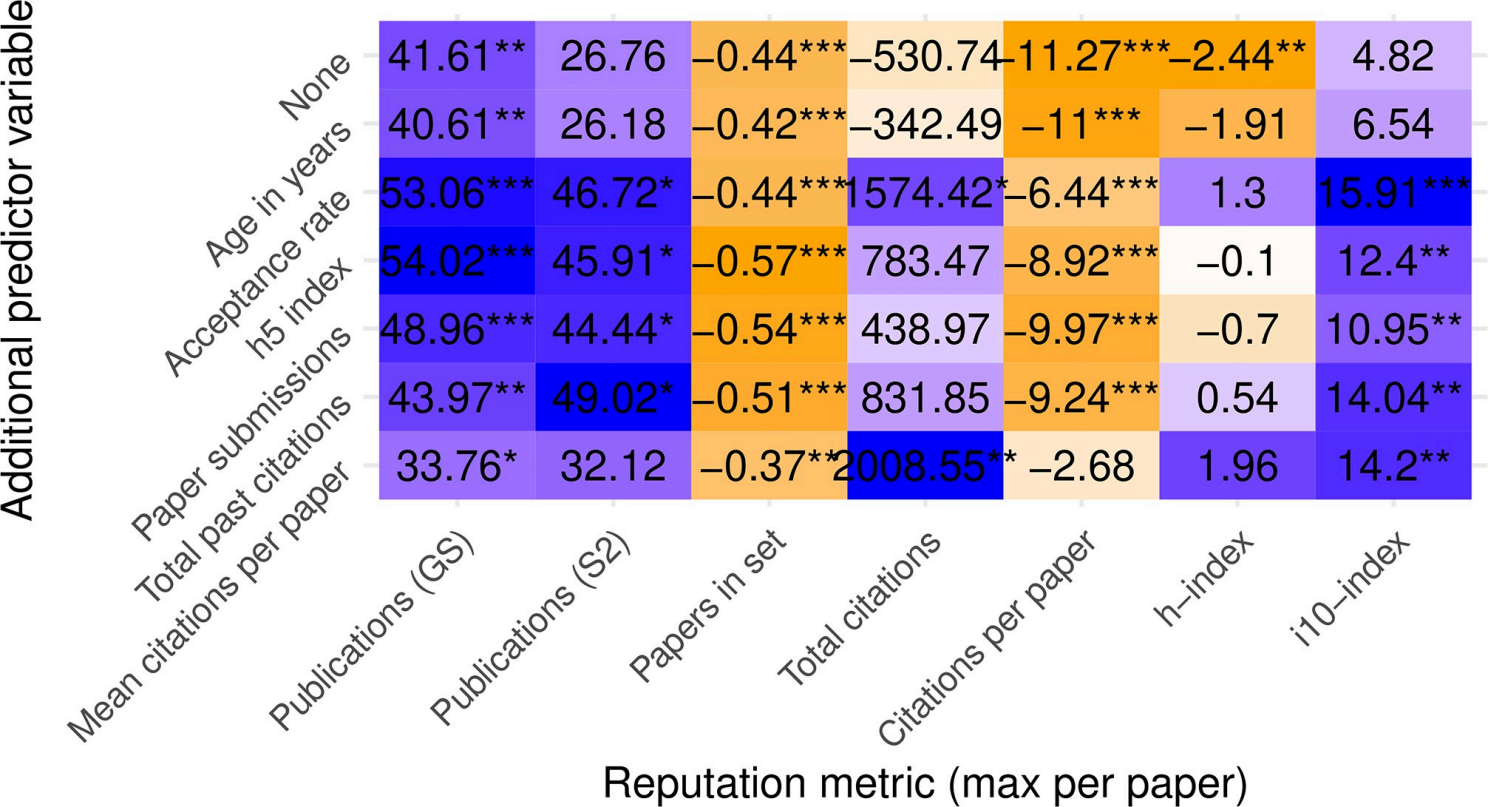
Linear regression of various author prestige metrics as predicted by review policy and other conference variables. Values shown are the coefficient of the reviewing policy variable in the linear model and their associated p-values. Positive coefficients (purple) indicate higher reputation in SB conferences. Negative coefficients (orange) indicate higher reputation in DB conferences. Darker colors reflect larger-magnitude values, normalized by columns. Stars indicate p values below 0.05, 0.01, and 0.001, respectively.

To evaluate the effect of these confounding variables, we built fixed-effects and mixed-effects linear regression models with author prestige as the outcome variable (maximum in paper), an indicator variable for reviewing policy (where 1 = SB, 0 = DB) as the fixed effect predictor variable, and conference as a random effect in the mixed-effects model. The first row of Fig 8 parallels the first row in Fig 3 and shows the coefficient of the reviewing policy alone on author prestige, which often exhibits a statistically strong association. Each row afterwards includes one of the variables listed above as an additional fixed effect that could stand in for conference prestige.

**Fig 9:**
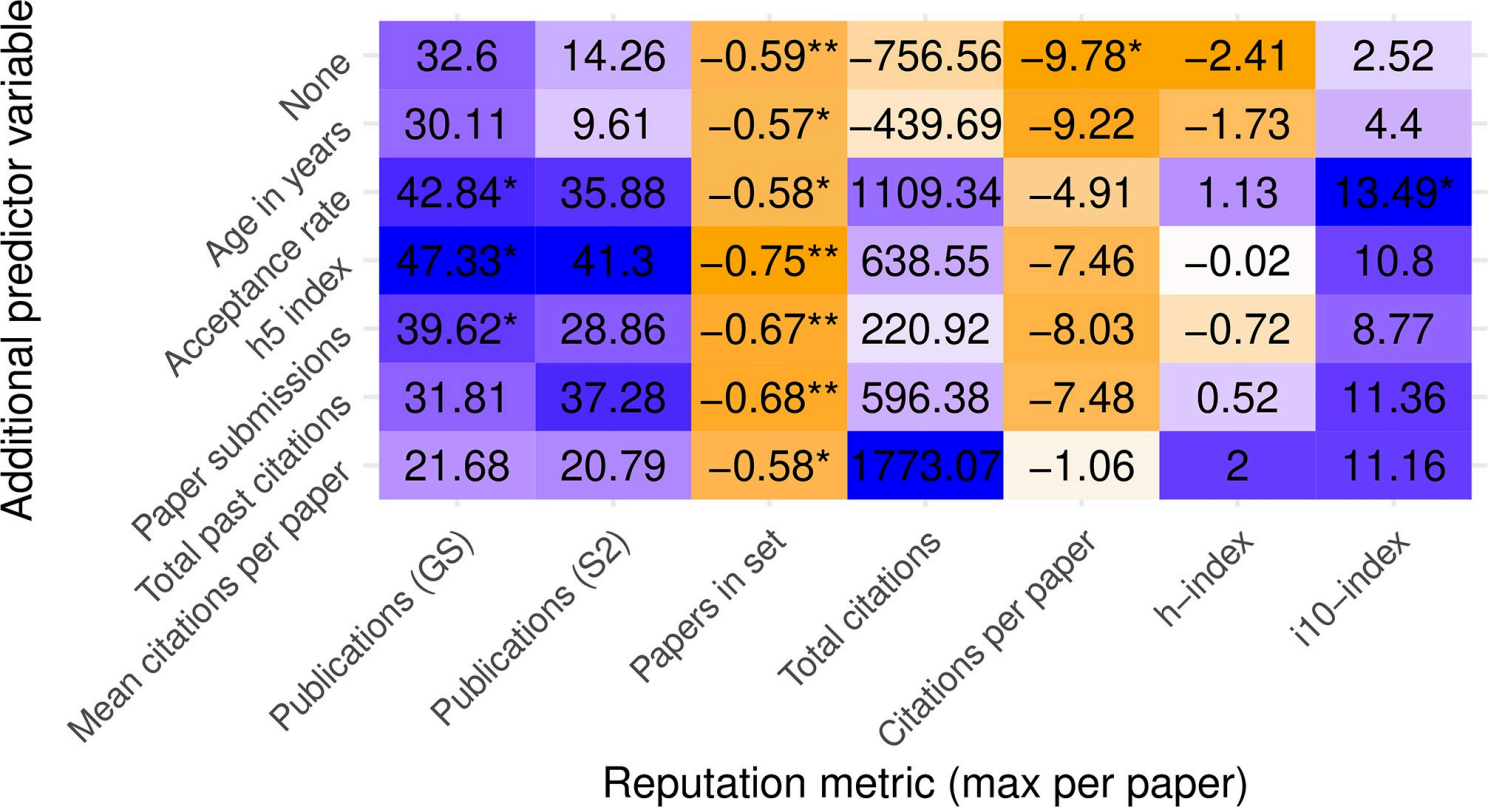
Mixed-effects linear regression models of various author prestige metrics as predicted by review policy and other conference variables with conference as a random effect. Values shown are the coefficient of the reviewing policy variable in the linear model and their associated p-values, estimated via Satterthwaite’s degrees of freedom method [30]. Positive coefficients (purple) indicate higher reputation in SB conferences. Negative coefficients (orange) indicate higher reputation in DB conferences. Darker colors reflect larger-magnitude values, normalized by columns. Stars indicate p values below 0.05, 0.01, and 0.001, respectively.

All of the analyses conducted up to the linear regression model assume the papers are independent observations. However, we believe papers in the same conference are likely to be more correlated, and therefore we account for this correlation explicitly in the linear mixed-model (Fig 9). In comparing Figs 8 and 9, notice that the significance of the reviewing policy is greatly dampened when accounting for conference. For the rest of this section, we focus on the mixed-effects model.

Looking first at citation-based author metrics (total citations, citations per paper, and h-index), we can indeed confirm that adding a conference prestige confounder and accounting for the intraclass correlation within conferences generally lowers the values of the review-policy coefficient and its significance, sometimes even changing signs.

Some of these citation-based author-conference relationships are almost tautological in nature. For example, we can expect to find people with many total past citations publishing more in conferences with more total past citations, because it is likely that the same highly-cited papers that contributed to a person’s count also contributed to the conference’s count, assuming some consistency in publishing venues over time. Such strong associations obviate other explanatory variables such as review policy, which is why the coefficient and significance of reviewing policy drops to near-zero when we correct for conference prestige.

The picture becomes less clear when we look at the first three columns, representing publication-based author-prestige metrics. They suggest that adding an explanatory variable does little to change the direction of prestige bias evidence: total past papers are still indicating higher prestige in SB conferences and total current papers are still indicating higher prestige in DB conferences.

We believe that the explanation for this apparent contradiction lies in the competitive nature of citation-based metrics. For publication-based metrics, it is harder to draw the same conclusions. There is little reason to assume that a person with many past or present publications also has a history of publishing in more competitive conferences. If anything, we would expect some prolific authors to publish less in conferences with a low acceptance rate, simply by making a probabilistic argument. In that case, we would also expect to see even higher values for the reviewing policy coefficient when correcting for low-prestige conferences, as some of the rows indeed appear to show.

As for the last column (i10-index), it appears to behave more like a publication-based metric than a citation-based metric. Although this metric incorporates both metrics, we believe it is influenced more by publication count than by their “impact,” because the “impact” threshold to inclusion in this index, namely 10 citations, is not particularly restrictive.

In summary, even the effect of correction for confounding variables appears to be sensitive to the metrics chosen. the inclusion alone of confounding variables could weaken any observed association between reviewing policy and author prestige for “impact-based” metrics and strengthen it for “productivity-based” metrics. Every one of the conference variables we tested could potentially serve as a better predictor of author reputation than reviewing policy, because of their strong correlations.

#### Logistic regression

Next, we revisit the treatment of an author’s prestige as a categorical variable, rather than a continuous variable. If we treat fame as a Boolean factor instead, i.e., an author’s name is either recognized or not, then we can repeat our analysis with mixed-effects logistic regression. We can do so by picking a “fame threshold,” above which we consider an author’s name to be recognized. As discussed earlier, senior (more experienced) authors show the same preference for DB or SB conferences regardless of the threshold selected to define seniority, at least for h-index and publication count. We will therefore use an arbitrary binary threshold of “top 10%” for each reputation metric to classify those authors who are most likely to be recognized.

**Fig 10:**
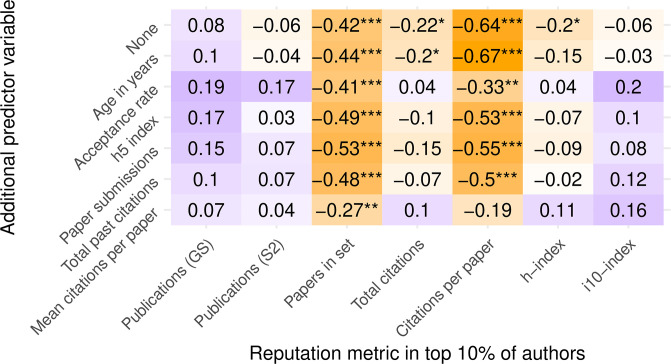
Logistic regression of the top decile of various author prestige metrics as predicted by review policy and other conference variables. Values shown are the coefficient of DB reviewing in the logistic model and their associated p-values. Positive coefficients (purple) indicate higher reputation in SB conferences. Negative coefficients (orange) indicate higher reputation in DB conferences. Darker colors reflect larger-magnitude values. Stars indicate p values below 0.05, 0.01, and 0.001, respectively.

**Fig 11:**
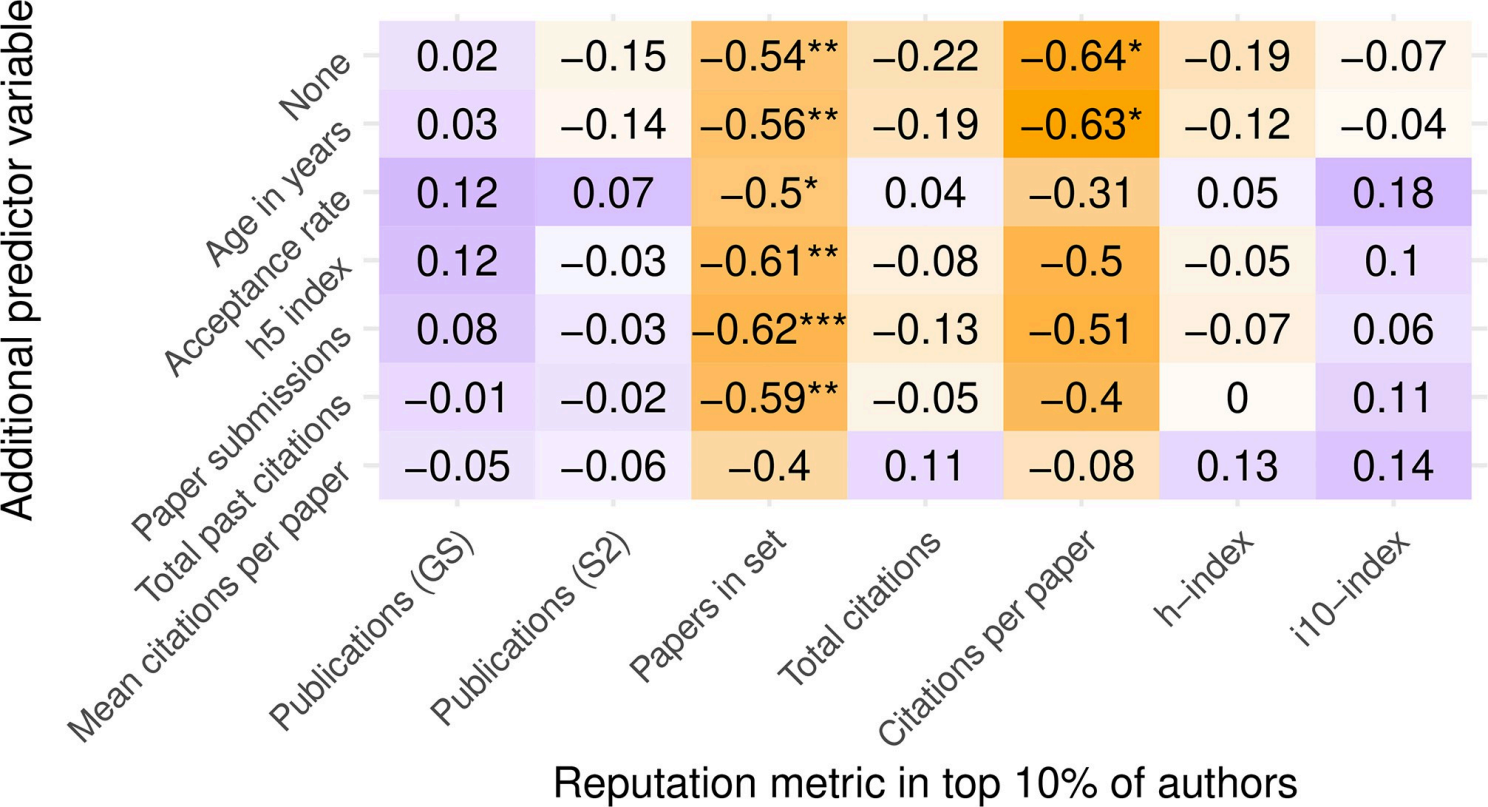
Mixed effects logistic regression of the top decile of various author prestige metrics as predicted by review policy and other conference variables with conference as a random effect. Values shown are the coefficient of DB reviewing in the logistic model and their associated p-values, estimated via Satterthwaite’s degrees of freedom method [30]. Positive coefficients (purple) indicate higher reputation in SB conferences. Negative coefficients (orange) indicate higher reputation in DB conferences. Darker colors reflect larger-magnitude values. Stars indicate p values below 0.05, 0.01, and 0.001, respectively.

Figs 10 and 11 depict the effects of this binary treatment of author prestige on the association with reviewing policy, corrected for conference prestige. Directionally, the results are similar to those of the linear regression. However, restricting name recognition to a specific quantile adds two desirable properties to the coefficient comparison: it normalizes all the coefficients to the same range from 0 to 1, and eliminates the outsized effect of high-prestige author outliers on the linear regression. Consequently, observing general trends becomes easier and less noisy.

The most noticeable general trend with this binary classification is that most coefficients are now statistically nonsignificant, meaning that it is now harder to claim prestige bias one way or the other, regardless of confounding variables. The two columns that show significant results are papers-in-set and mean-past-citations-per-paper. Both metrics show the same direction (higher reputation in DB conferences) and the significance of this preference does not abate with confounding conference factors.

We cannot offer a satisfying explanation to why these two metrics stand out this way, nor can we fully explain why the other columns lost their statistical significance in the binary treatment, except for the dampening of outliers. We believe that the main takeaway from this additional perspective and its perplexing results, as has been noted repeatedly throughout this study, is that the observation of prestige bias is highly sensitive to our choices of metrics. It could also be sensitive to the methodology for cleaning and preparing the raw data for analysis, especially in the presence of outliers and suspicious observations, which is the topic we address next.

### Handling missing and suspect observations

To complete our experimental evaluation, we consider the effects of *data cleaning*. Preparing this dataset for analysis required a thorough exploration of each variable and observations for missing or suspect values. This process does not follow a formal set of rules but instead usually requires the researchers to make several subjective choices, each of which potentially impacts the final results. In this subsection, we explore how the handling of missing and suspect observations potentially impacts conclusions of prestige bias.

Fig 1 displays the distributions of the various author-level prestige metrics. All metrics are right skewed and there are reasons to suspect the quality of the values in both the upper and lower tails of these distributions.

On the upper-end tails, reputation metrics are likely inflated because of name disambiguation issues, i.e., attributing too many publications to certain names who may be shared across different researchers. Previously, we explored a few analysis techniques that mitigated the effect of outliers, such as the Wilcoxon Signed Rank Test and a binary classification of fame, to see if they changed the results. For a given metric and aggregation technique, the conclusions regarding prestige bias were similar between these outlier-resistant measures and the other techniques, suggesting a limited effect for these inflated data. We therefore chose not to winsorize these inflated metrics, a common data cleaning technique for highly right-skewed data.

On the lower tail, note that 30.72% of the values for the GS measures are missing for authors for whom we could not uniquely identify a GS profile. Proportionally, these authors are distributed almost evenly between SB and DB publications. Since the median number of publications from S2 is only NA for those without GS measures—compared to NA for those with GS measures—these authors with missing values are likely less experienced and therefore would contribute to the lower tail of the author-level prestige metric distributions. The variation in S2 publications between those with and without GS profiles provides evidence that the missing values are not missing at random and that we should determine how sensitive our results are to how the missingness is treated.

For handling these missing values, we consider three strategies. The simplest approach, which we have used in all previous experiments, involves removing the missing values when computing aggregate measures. This strategy likely inflates some paper aggregation metrics such as the mean, but has less influence on the maximum metric.

On the opposite end of the spectrum, we also considered imputing the missing metrics with the minimum value for that metric. This imputation assumes that the missing values all represent new researchers and will pull down the mean paper aggregation metrics, but again will hardly influence the maximum metric.

**Fig 12:**
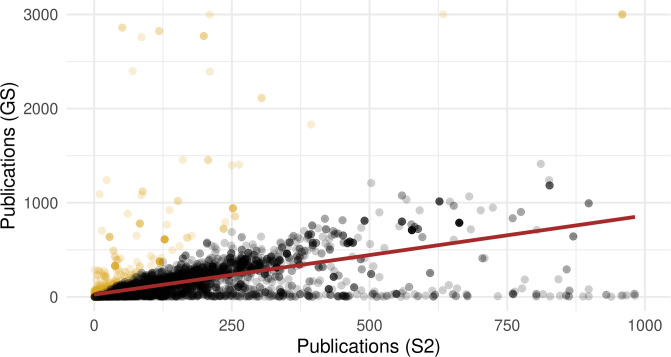
Scatterplot of the relationship between the two author-level, total publication metrics. The estimated linear regression line used for imputation is included in black and pointed where the GS metric is 3 times the S2 metric are highlighted in yellow.

Lastly, we considered imputing the missing values based on a linear regression model built on the total publications from S2, which is moderately correlated with the GS metrics (Fig 2). For the linear imputation, we also imputed the GS metrics if the ratio of the total publications from GS compared to S2 was greater than 3, which indicated a potential inflation of the values of GS metrics for those authors (Fig 12). Similarly, to minimize imputing inflated GS metrics, we winsorized at 1000 the very heavy tail of the total publications from S2 before computing the imputed values.

**Fig 13:**
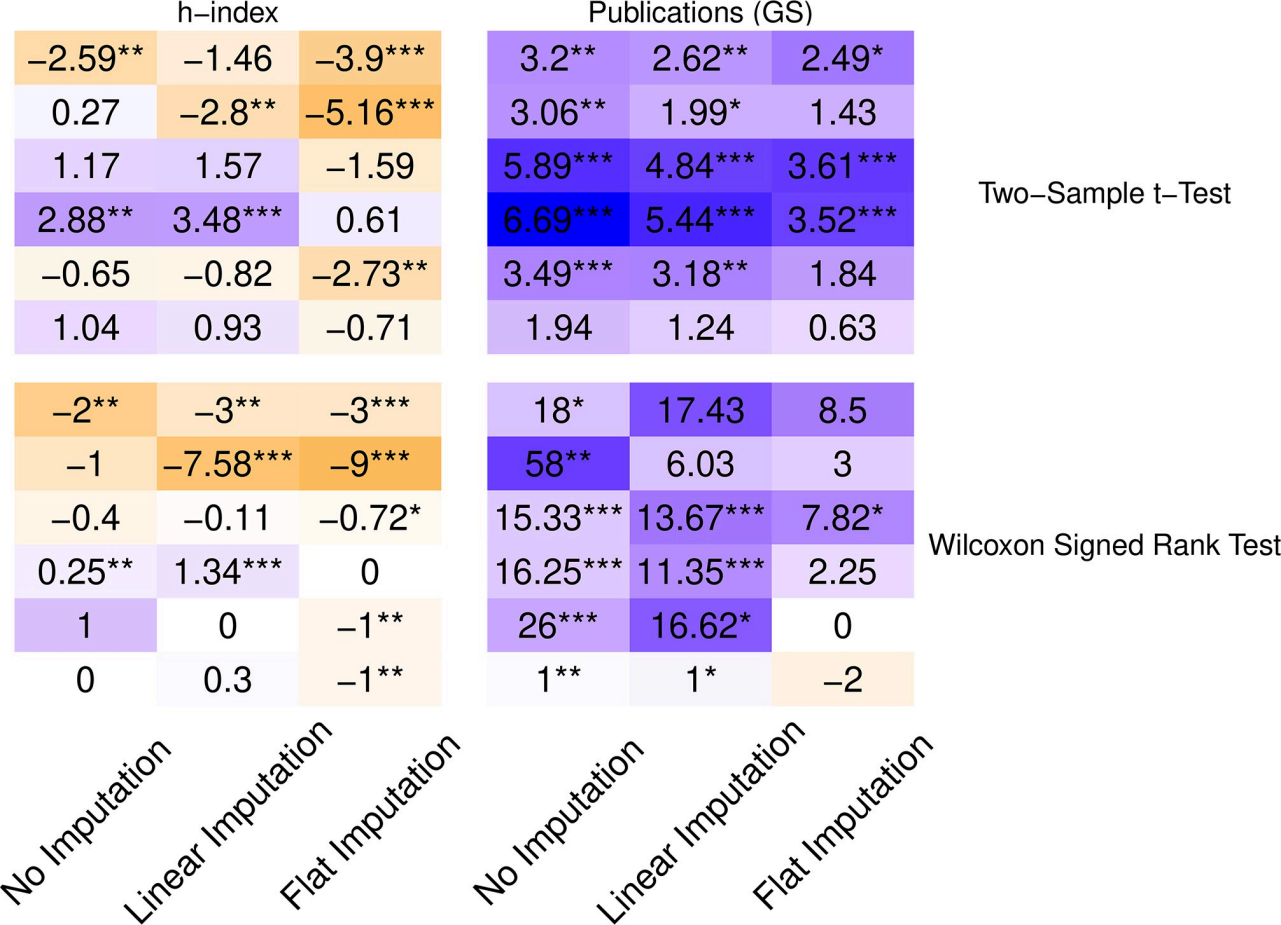
Test statistics of the Student’s t-test and the difference in medians for the Wilcoxon test for publications (GS) and h-index, aggregated across papers for three strategies of handling missing values. Positive values (purple) indicate higher reputation in SB conferences. Darker colors reflect larger-magnitude values, normalized by columns and test method. Negative values (orange) indicate higher reputation in DB conferences. Stars indicate p values below 0.05, 0.01, and 0.001, respectively.

Across these three strategies, the sign of the relationship between review policy and the prestige metric stays the same for most aggregations, though the significance of the results does vary (Fig 13). The linear imputation method acts as a compromise by typically providing a test statistic and p-value that is between those provided by the other two methods. Generally, it appears that imputing the missing data has little impact on prestige-bias analysis because most of the missing data are for the less-experienced authors, who do not impact many of our aggregation metrics. However, researchers must remain cognizant of these choices and transparent about how they handle missing values.

## Discussion

The previous section explored the observed relationships between author prestige and review policy across numerous dimensions. We started with a simple model for prestige bias, as many studies do, and then one by one analyzed the effect of the assumptions underlying the model. Our analysis compared dozens of parameter combinations, which poses the risks of data mining and “p-hacking”. We therefore suggest not to ascribe too much meaning to the individual p-values reported here, but rather to look at the bigger question: how is it that different perspectives on the same data can support diametrically opposed conclusions?

For example, we have seen that different reputation metrics can yield contradictory results. Even a single popular metric, number of publications, can yield very different distributions based on the database it comes from, because each database comes up with its own choices to inherently hard questions like “what constitutes a publication?” and “how to disambiguate names?”.

The most striking metric contrast surfaces when comparing number of publications to h-index, even from the same data source (GS). Under most treatments, famous authors by number of publications appear to associate strongly with SB conferences in our dataset, where the opposite is true for famous authors by h-index. Since these two metrics are widely used, it is worth investigating the source of the discrepancy, as a case study for the process of untangling these contradictions.

**Fig 14:**
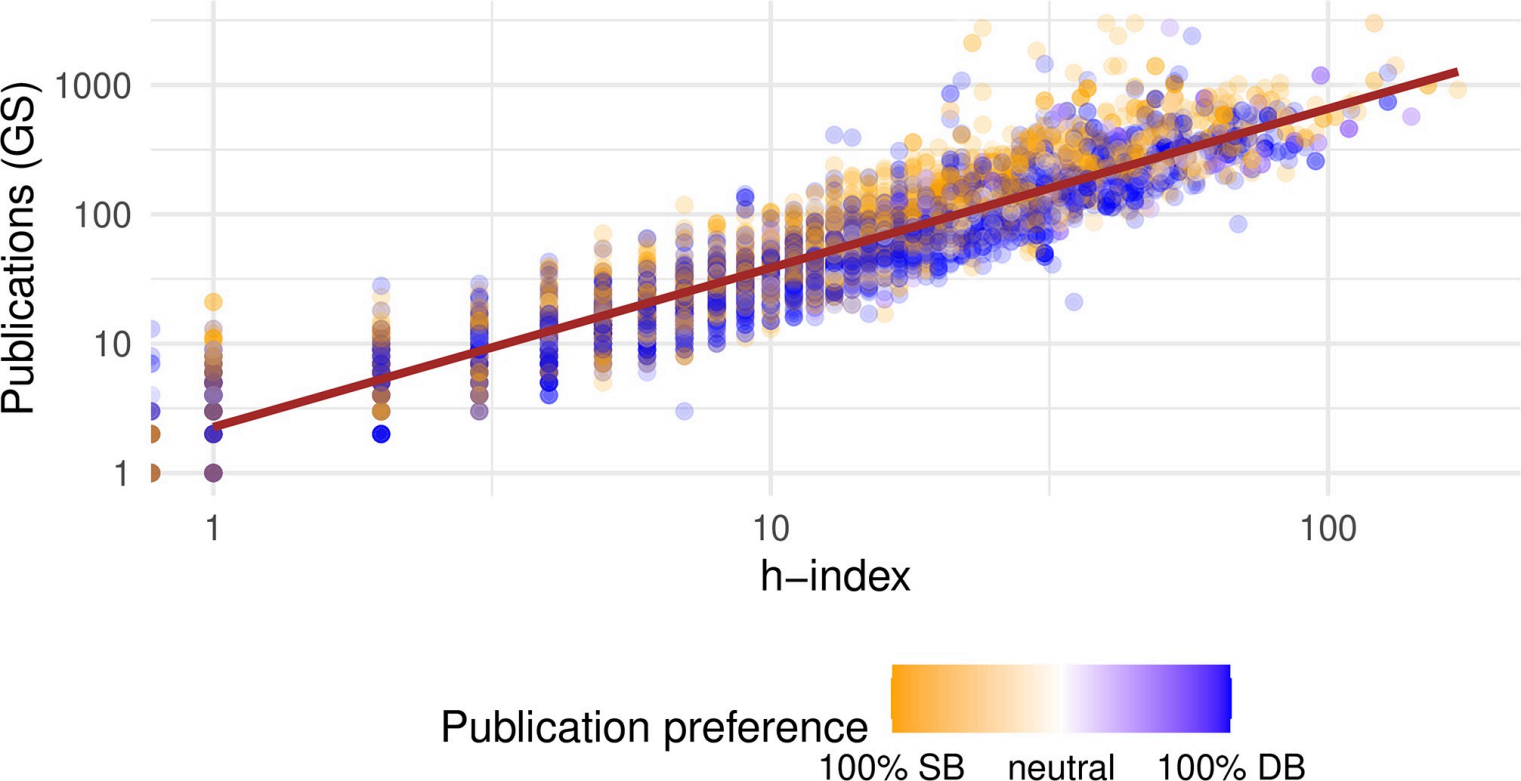
Scatterplot of the relationship between number of publications (GS) and and h-index for all authors with data (log scale). Color denotes the ratio of an author’s papers from single-blind conferences in this dataset. Brown line denotes linear regression of all data points.

Fig 14 compares these metrics across all authors with GS data, with the color of each point representing the percentage of papers in our set that each person published in SB or DB conferences. Despite the strong correlation between the two metrics, it is clear that they do not always agree. Some researchers exhibit relatively high h-index and some exhibit relatively high publication count, compared to the average population as represented by the regression line.

Observe that the authors below the regression line (relatively high h-index) tend to publish more often in DB conference, whereas the opposite is true for authors with relatively many publications. This implied dichotomy suggests two broad types of researchers, which we dub as “impact” and “prolificity”, respectively. Double-blind conferences probably attract more of the “impact”-type researchers because these conferences are also associated with higher reputation metrics, including citations (Table 1). On the other hand, “prolificity”-type researchers probably publish more in the SB, less-competitive conferences, which would statistically lead to more accepted papers but likely also to a lower relative h-index. As another data point for venue preference, early-career researchers were found in a related study to actually prefer higher-impact journals and were influential in the team’s choice of where to publish [31].

We cannot demonstrate a decisive difference in submission preference without data on all submitted papers. However, we can find some confirmation in a different, retroactive statistic: how well-cited are the papers in our dataset, now that a few years have passed since publication. If we look at GS statistics for each paper exactly 36 months after it was published, we find that DB papers average 39.6 citations per paper, significantly higher than SB’s 16.9 (*t* = 8.05, *p* < 10^−9^). The difference in medians, 13, is also quite significant (*W* = 1075516.5, *p* < 10^−9^). These statistics provide additional evidence that DB conferences in our dataset are the more prestigious ones, and support the notion that “impact”-type researchers would choose to publish in them. It also lends additional support to the idea that prestige-bias evaluation should account for venue prestige when comparing across venues or across years, for example, using a mixed-effects model. Indeed, when we attempt to control for conference prestige using the proxy of mean citations per paper, most of the previously found differences in bias lose statistical significance (Fig 9).

## Related work

### Prestige bias in peer review

The integrity of the peer-review process is of utmost importance to science and scientists, and is thus an active research area all of its own. As an inherently human process, influenced by the opinions, perspective, and understanding of human reviewers, peer review is potentially subject to various cognitive biases. Numerous studies attempt to identify, measure, and offer interventions for such biases. However, the gold standard for such experiments, randomized controlled trials, is exceedingly difficult to carry out and generalize [32–35]. Aside from the usual ethical challenges of experimenting on humans, the properties of the scientific publication make repeatable and controllable experiments particularly difficult. Papers are typically disallowed from concurrent submission, so A/B experimentation with the same papers is both complex and only partially controlled. Moreover, actual scientific publications have potentially enormous impact on everyone involved, so the review process cannot be arbitrarily tweaked for the purposes of experimentation.

The upshot of such constraints is that there are relatively few studies on peer-review bias using representative controlled experiments, they are often limited in scope to one journal or conference, and depending on their data and methods, can reach incompatible conclusions [3, 36, 37]. A literature survey from 15 years ago on the question of prestige bias found mixed results [38], as have we in the literature since then.

In one famous study, for example, Fisher et al. performed a randomized trial on 57 consecutive submissions to the Journal of Development and Behavioral Pediatrics. Each paper received 2 SB and 2 DB reviews, and acceptance decisions were compared to the number of publications of each paper’s lead author and most-experienced author. The paper concludes that SB reviewing favors authors with higher publication counts.

A similar but more recent example experimented on the submissions to the WSDM’17 conference on data mining [3]. The experiment split the PC into two halves, one using SB reviewing and the other DB, with two people from each PC reviewing each paper. The study looked at the following covariates: author gender; sector of majority coauthors per paper; most common country of majority coauthors; country homophily with reviewers; famous author (based on a criterion of at least 100 past publications, 3 of which in WSDM); and affiliation prestige (based on top-50 university list or top-4 company list). Their findings suggest a strong bias in favor of authors with individual or affiliation prestige in SB reviews. The paper also acknowledges the difficulty and rarity of such controlled experiments and points out to a single other comparable study [4]. As Tomkins et al. summarize:

Perhaps the best-known experimental study of SB vs. DB reviewing behavior, and to our knowledge the only controlled experiment in this area other than our own, is the study by Rebecca Blank (15). Over several years, 1,498 papers were randomly assigned to SB vs. DB reviewing condition. While Blank performs detailed analyses of many facets of the data, we may summarize part of the high-level findings as follows. First, authors at top or bottom institutions do not see significant differences in acceptance decisions based on reviewing model, but authors at midtier institutions perform better in a SB setting, as do foreign authors and those out-side academia. Second, there is a mild indication, not statistically significant, that women do slightly better in DB review.

[3]

A different experimental approach, using a fabricated article listing two famous researchers as authors, found that “Reviewers were more likely to recommend acceptance when the prestigious authors’ names and institutions were visible… than when they were redacted… and also gave higher ratings for the methods and other categories” [9].

Yet another approach, with controversial ethical ramifications, was reported in a study that retitled and resubmitted 12 famous papers in psychology with fictitious authors and institutes [10]. Three of those were detected as resubmissions, and eight of the remaining nine were rejected in SB peer review.

Closer to our field of study, Madden and DeWitt [27] looked retrospectively at VLDB and SIGMOD conference papers (SIGMOD is also in our dataset). Similar to our approach, this observational study did not look at rejected papers or experiment with the review process itself. While our study compares the publication ratios of famous authors across SB/DB conferences in the same field and point in time, this study compares them across time for the same two conferences, before and after they switched from SB to DB reviewing. For the purpose of their study, “famous authors” were defined as those 25 “prolific” individuals with at least 20 past publications in these two conferences.

Interestingly, a followup analysis on the same dataset came to a contrary conclusion by simply comparing median publication rates instead of means [28]. The justification for using medians was that it is more robust to outliers, which for this dataset was particularly relevant because only one of the measured values fell above the mean. This study not only demonstrates the sensitivity of the conclusions to the metrics used, as we show as well, but also cautions that “there are probably a lot of other factors that must be taken into consideration before the database community makes a final choice on whether to continue with double blind review.”

### Prestige metrics

Since we found no consensus in the literature on the existence of prestige bias in SB reviewing, or even on the methods to measure it, could we at least find a common answer to the smaller question, “how should we quantify research prestige?”

Unfortunately, we found no such answer either. The field of bibliometrics is rich with studies comparing different and contradictory metrics to evaluate the productivity, prestige, and impact of scientific work and researchers, and we can only review a fraction of this discussion in the scope of this paper.

The highly influential h-index, for example, was proposed to combine productivity with echo, and to address specific shortcomings of the publications and citations count metrics, such as sensitivity to a single highly-cited paper (“one-hit wonders”) and prolific authors in low-quality venues [29, 39, 40].

But some researchers argue that a few influential papers should count more than a bevy of poorly cited papers [40, 41] and that h-index is overly sensitive to the length of a researcher’s career [42].

Furthermore, the h-index has additional shortcomings of its own. It can be inconsistent [43], or be manipulated with self-citations [44]. It also does not account properly for the magnitude of individual contributions in team papers [26, 40]. This criticism applies to the total publications metric as well, and most other widely used reputation metrics. Finally, even when deciding to use h-index, several studies found different counts in different databases (as have we). They caution that more than one source should be used to compute h-index accurately, and that comparisons between researchers should be limited to the same data source [45, 46].

Other citation-based reputation metrics have been proposed to overcome some of these limitations, such as g-index [41], p-index [47], I3 [48], AR-index [49], and even simply total citations. All of these metrics have been found to correlate with each other (as we have also observed) and are generally sensitive to changes in number of publications as well [50, 51]. In fact, Bertoli-Barsotti and Lando built a model to predict one reputation metric based on the others, which shows that the relationships between metrics can be complex and nonlinear [52]. These relationships and model mean that most metrics are codependent, so adding reputation metrics to evaluate bias may not add as much signal as desired.

Nevertheless, we believe that it would be wrong to focus on a single metric without performing a sensitivity analysis with additional metrics that may provide counter-evidence. Carpenter et al. also concluded, based on a comparison of traditional and emerging publication metrics, that no single metric is descriptive enough [24]. Instead, they suggest that a complete picture of author reputation includes multiple metrics, including traditional productivity- and impact-level metrics, as well as document-level metrics such as views, downloads, tweets, and translations. Standardizing and incorporating such *altmetrics* as part of an author’s reputation or name recognition could potentially improve the analyses of prestige bias in peer review.

## Conclusions

This study focuses on the topic of prestige bias in peer review. It analyzes an expansive dataset from one large subfield of computer science, and examines multiple pieces of statistical evidence on the association between “famous authors” and higher publication rates in single- or double-blind conferences. Rather than focusing on identifying conclusive evidence one way or the other, which our dataset is too limited to offer, we focus instead on the methodology of this search, and the many choices involved in such research.

Throughout our empirical results, we explore the decisions and assumptions that are involved in prestige-bias analysis, both for experimental and observational studies. These choices can all make a substantive difference in the direction and significance of the study’s conclusions, and include:

- choice of reputation metric;
- reputation aggregation methods across coauthors;
- continuous or categorical treatment of reputation;
- confounding variables and mixed-effect models;
- handling of missing and suspect observations.

Perhaps the most critical of these dimensions is the choice of reputation metric. This choice is a central deciding factor in whether our dataset yields bias consistent with review bias. The two most common metrics, number of publications and h-index, produced statistically significant contradictory results on the same data. Our analysis reveals that the reasons for this apparent contradiction relate to the “type” of publication activities characteristic of different researchers. Some researchers appear to favor publishing a smaller number of highly competitive papers, some prefer to publish prolifically in less competitive venues, and others lie in the spectrum between these two groups. These types of researchers exhibit different metric values, and two established researchers could represent extreme opposites across these two common metrics.

This phenomenon complicates any analysis of prestige bias that uses a single metric for prestige, as most do, because it may misclassify some authors as “not famous” if they exhibit a higher metric value on the metric not chosen for the study. Cross-sectional observational studies are further complicated by the interaction of researcher type and confounding conference factors. The review policy of a conference is not an entirely independent random variable, and other conference factors likely play a larger role in the submission (and acceptance) decisions for a researcher. In our dataset, double-blind conferences tend to also be more competitive and prestigious. This property, which leads to higher citation-based metrics and lower publications-based metrics, likely attracts more researchers of the “impact” type.

Our main recommendation for future prestige-bias studies is therefore to not focus on a single reputation metric. More generally, we recommend that researchers design a thorough sensitivity analysis as part of their research, especially for observational data. The evidence for or against prestige bias appears to be strongly influenced by multiple factors (listed in this paper), and researchers could use these as a checklist to consider carefully before finalizing their conclusions. Experimental studies allow for more control and elimination of some confounding variables, like the publication venue. But even experimental studies still require careful evaluation of the question of defining “fame”, with important choices for metrics and aggregations.

We must also acknowledge that “reputation” and “name recognition” are not purely measurable values that lend themselves easily to quantitative analysis. Perhaps a better approach for experimental studies might be to ask reviewers to rank the “reputation” of the authors they reviewed, but only after all papers have been reviewed, to reduce priming effects. We hope further that this paper provides a guideline and a roadmap to studies on bias on how to carefully address methodological issues that could limit the generalization or credibility of their results.

## Supporting information

S1 Dataset Complete data and source code. (ZIP)

S1 Appendix Detailed conference list. (PDF)
