# Supplementary figures and images for: Metrics and methods in the evaluation of prestige bias in peer review: A case study in computer systems conferences
Source: PLoS One. 2022 Feb 25;17(2):e0264131. doi: 10.1371/journal.pone.0264131 (PMC8880855; doi:10.1371/journal.pone.0264131)

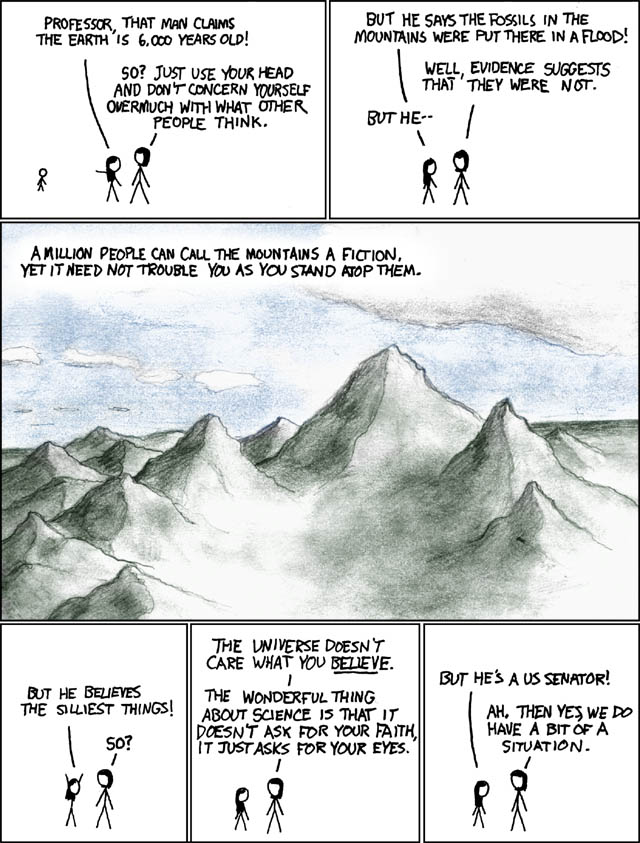

Supplement: S1 Dataset — (ZIP) [file pone.0264131.s001.zip › sysconf/docs/images/beliefs.jpg]

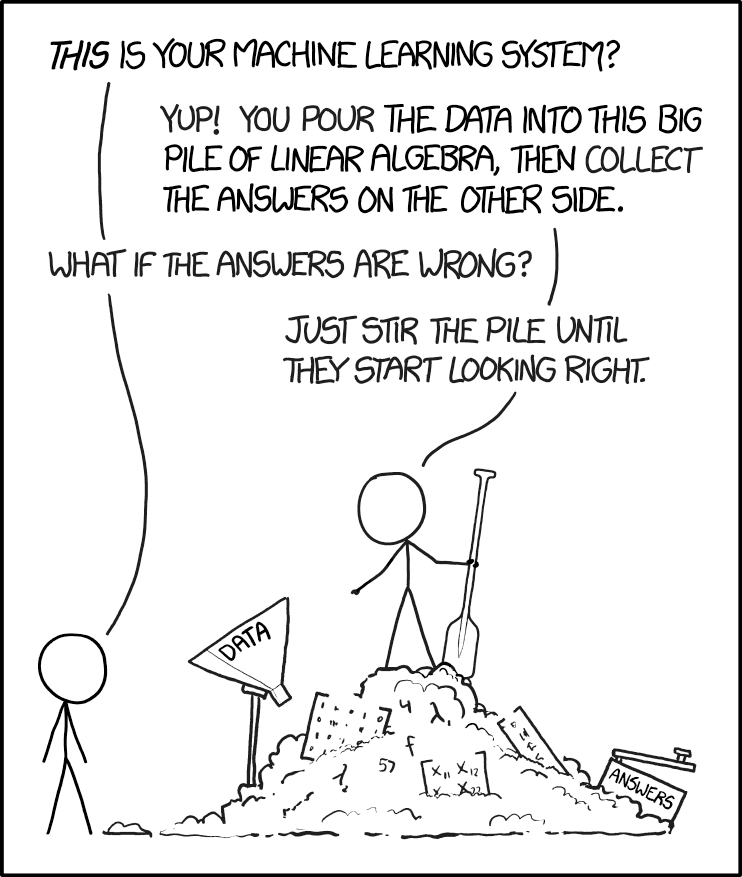

Supplement: S1 Dataset — (ZIP) [file pone.0264131.s001.zip › sysconf/docs/images/machine_learning_2x.png]

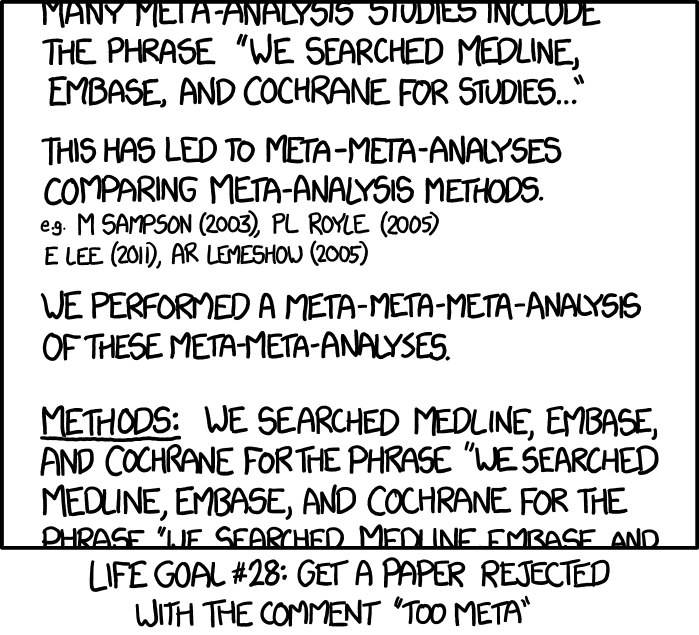

Supplement: S1 Dataset — (ZIP) [file pone.0264131.s001.zip › sysconf/pubs/web/images/meta-analysis_2x.png]

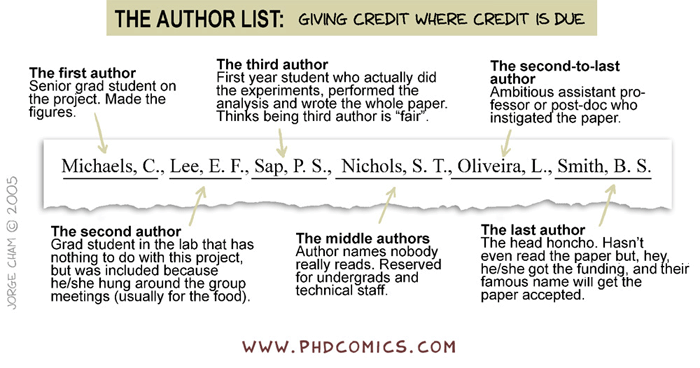

Supplement: S1 Dataset — (ZIP) [file pone.0264131.s001.zip › sysconf/pubs/web/images/phd031305s.gif]

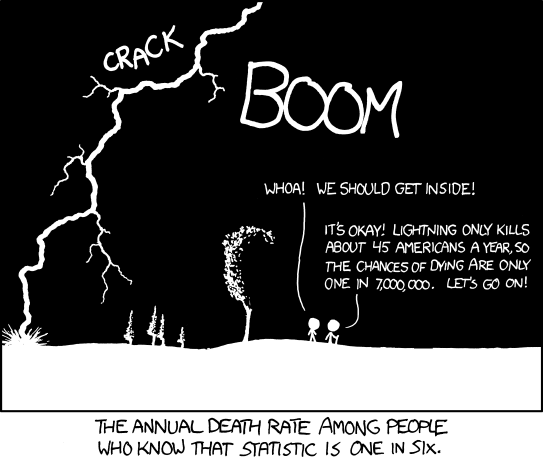

Supplement: S1 Dataset — (ZIP) [file pone.0264131.s001.zip › sysconf/pubs/web/images/conditional_risk.png]

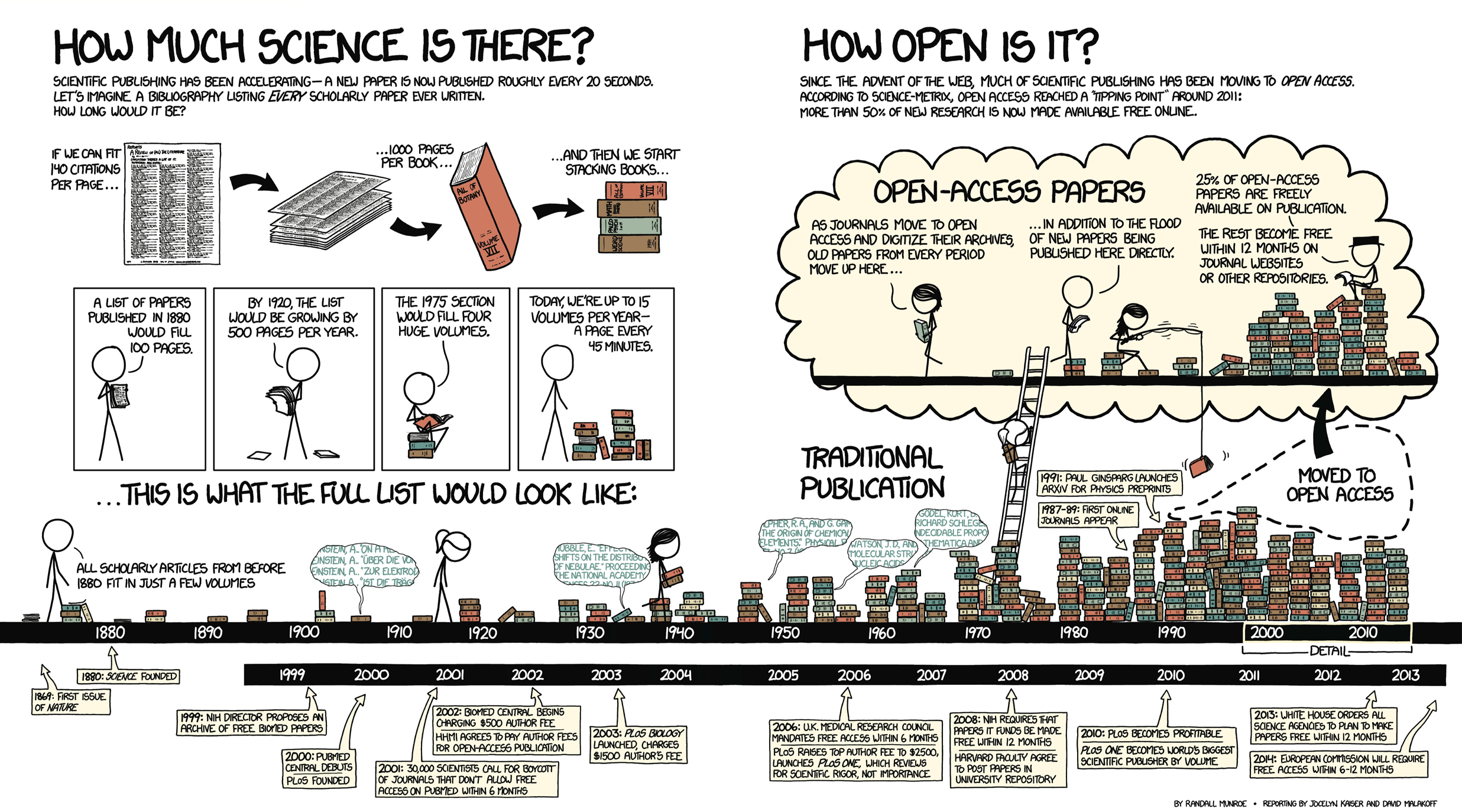

Supplement: S1 Dataset — (ZIP) [file pone.0264131.s001.zip › sysconf/pubs/web/images/infographic.jpg]

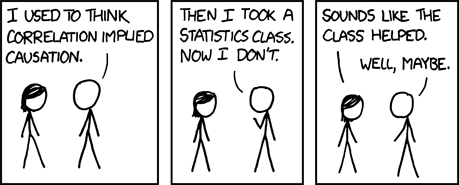

Supplement: S1 Dataset — (ZIP) [file pone.0264131.s001.zip › sysconf/pubs/web/images/correlation.png]

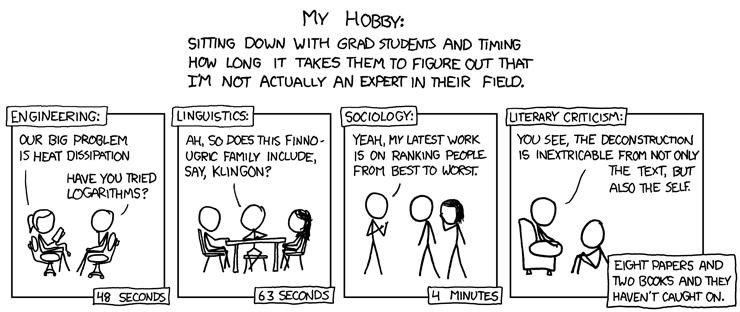

Supplement: S1 Dataset — (ZIP) [file pone.0264131.s001.zip › sysconf/pubs/web/images/impostor.png]

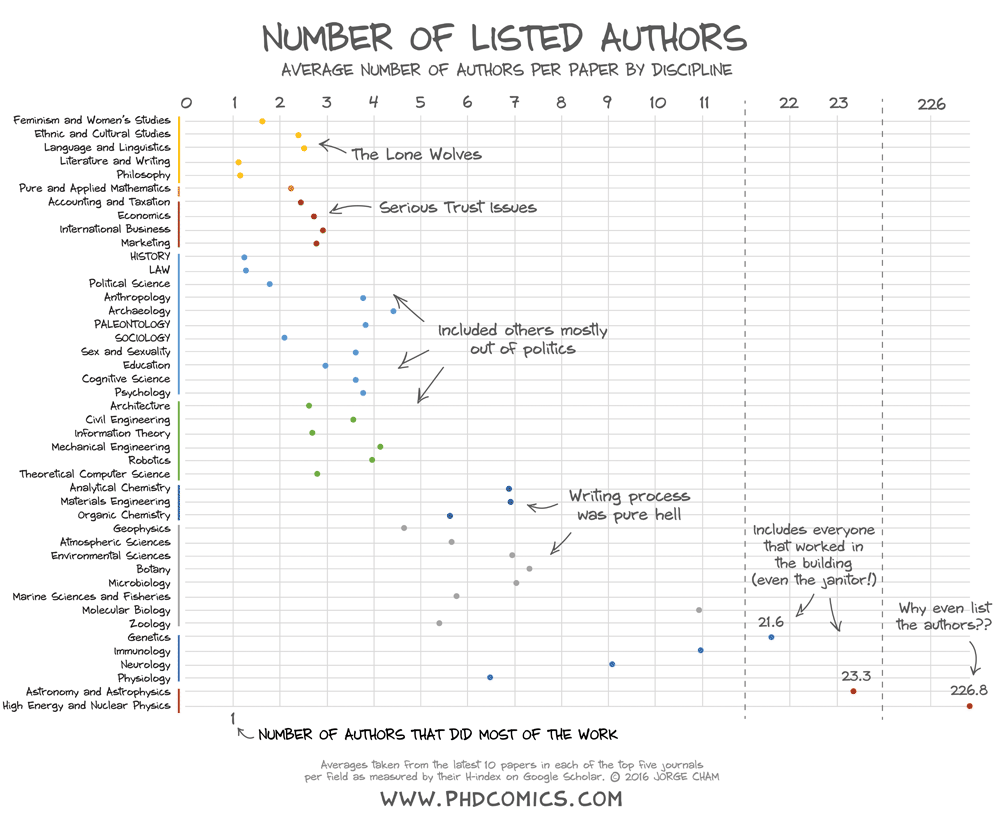

Supplement: S1 Dataset — (ZIP) [file pone.0264131.s001.zip › sysconf/pubs/web/images/phd120916s.gif]

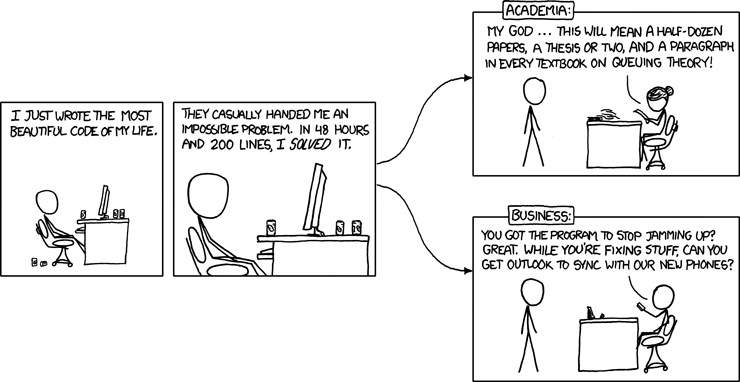

Supplement: S1 Dataset — (ZIP) [file pone.0264131.s001.zip › sysconf/pubs/web/images/academia_vs_business.png]

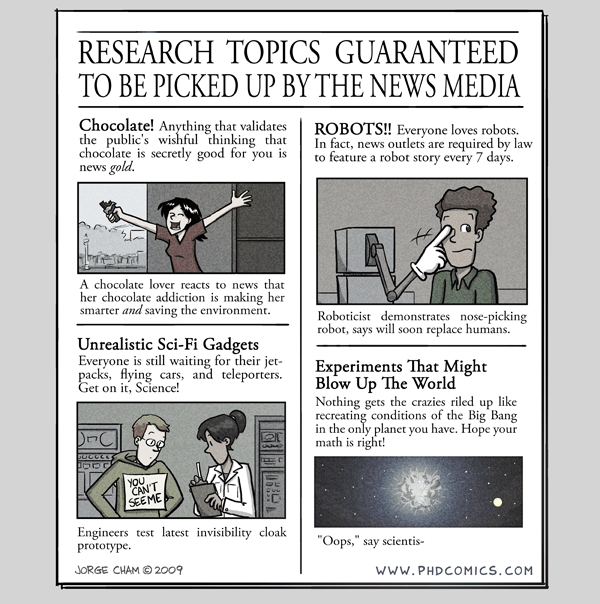

Supplement: S1 Dataset — (ZIP) [file pone.0264131.s001.zip › sysconf/pubs/web/images/phd052009s.gif]

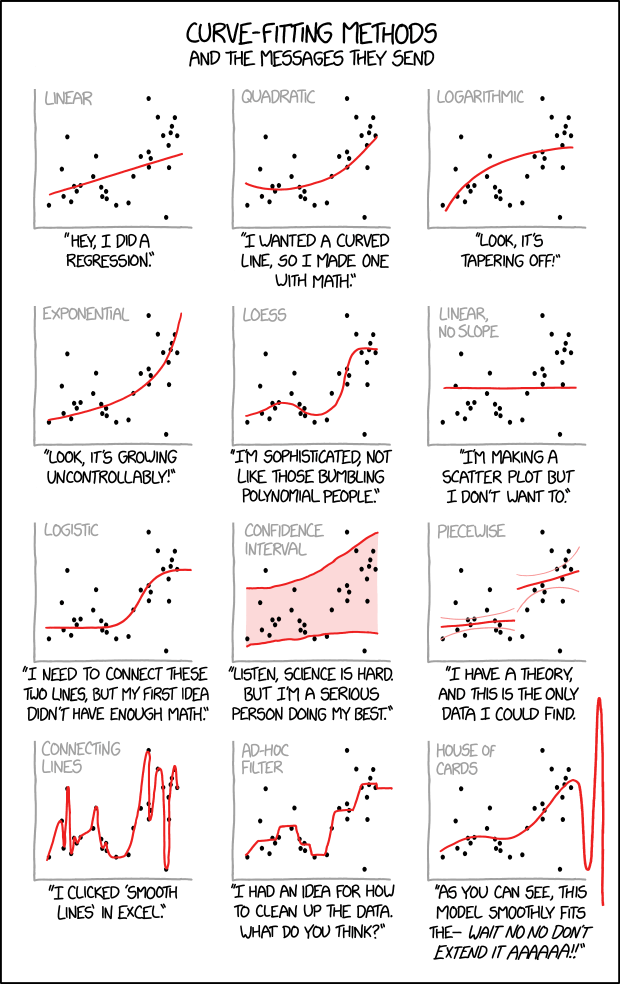

Supplement: S1 Dataset — (ZIP) [file pone.0264131.s001.zip › sysconf/pubs/web/images/curve_fitting.png]

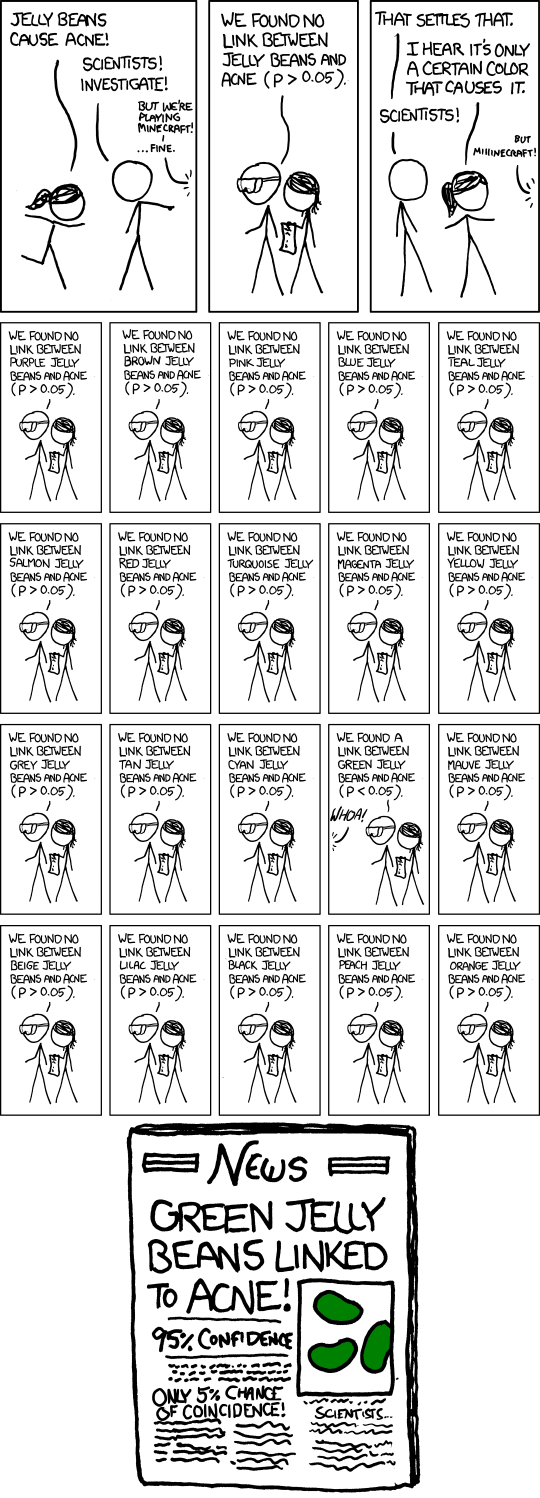

Supplement: S1 Dataset — (ZIP) [file pone.0264131.s001.zip › sysconf/pubs/web/images/significant.png]

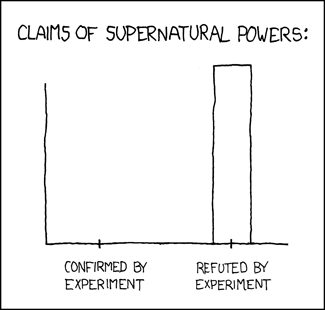

Supplement: S1 Dataset — (ZIP) [file pone.0264131.s001.zip › sysconf/pubs/web/images/the_data_so_far.png]

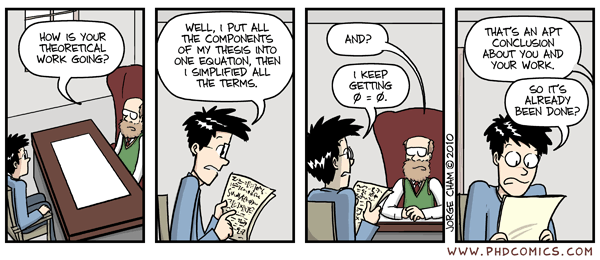

Supplement: S1 Dataset — (ZIP) [file pone.0264131.s001.zip › sysconf/pubs/web/images/phd110110s.gif]

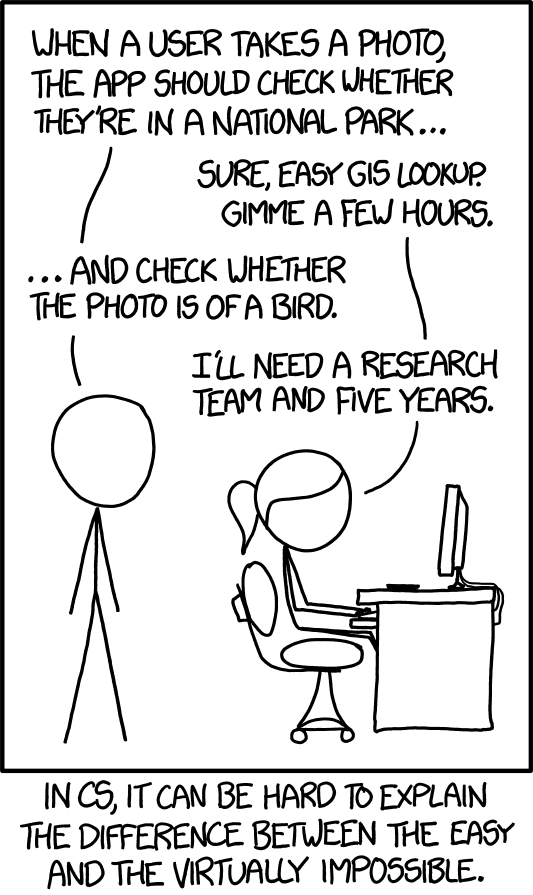

Supplement: S1 Dataset — (ZIP) [file pone.0264131.s001.zip › sysconf/pubs/web/images/tasks_2x.png]

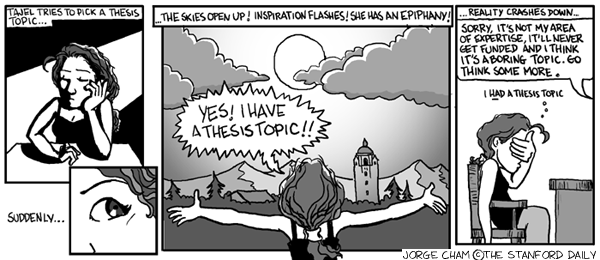

Supplement: S1 Dataset — (ZIP) [file pone.0264131.s001.zip › sysconf/pubs/web/images/phd100998s.gif]

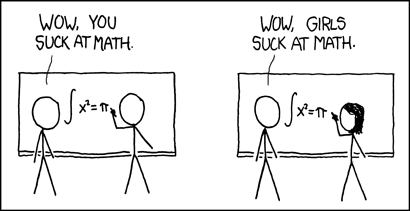

Supplement: S1 Dataset — (ZIP) [file pone.0264131.s001.zip › sysconf/pubs/web/images/how_it_works.png]

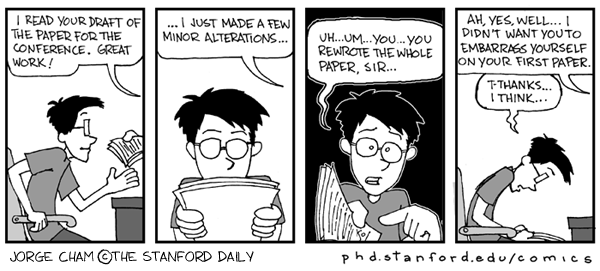

Supplement: S1 Dataset — (ZIP) [file pone.0264131.s001.zip › sysconf/pubs/web/images/phd010500s.gif]

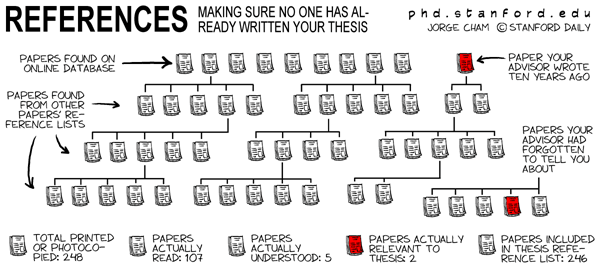

Supplement: S1 Dataset — (ZIP) [file pone.0264131.s001.zip › sysconf/pubs/web/images/phd022702s.gif]

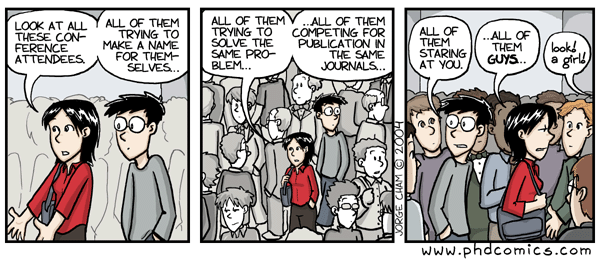

Supplement: S1 Dataset — (ZIP) [file pone.0264131.s001.zip › sysconf/pubs/web/images/phd081604s.gif]

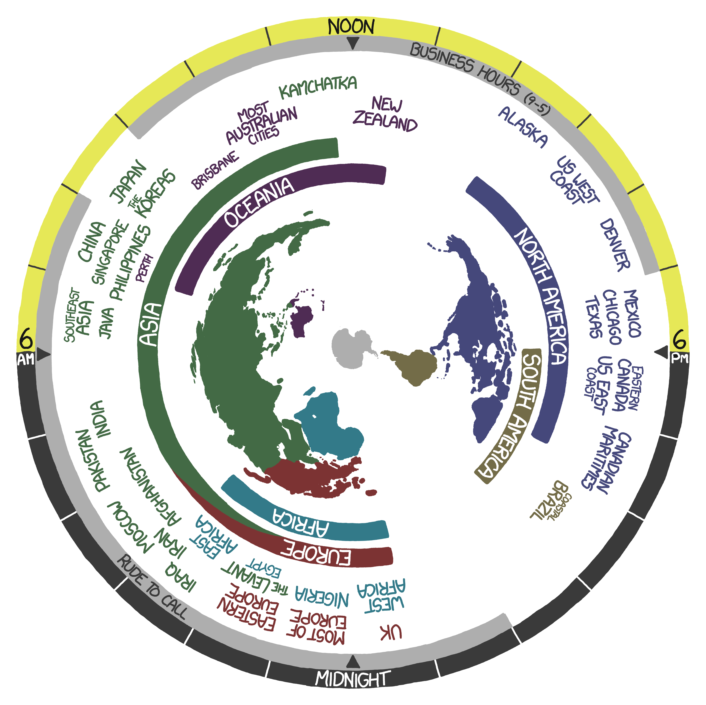

Supplement: S1 Dataset — (ZIP) [file pone.0264131.s001.zip › sysconf/pubs/web/images/11h30m.png]

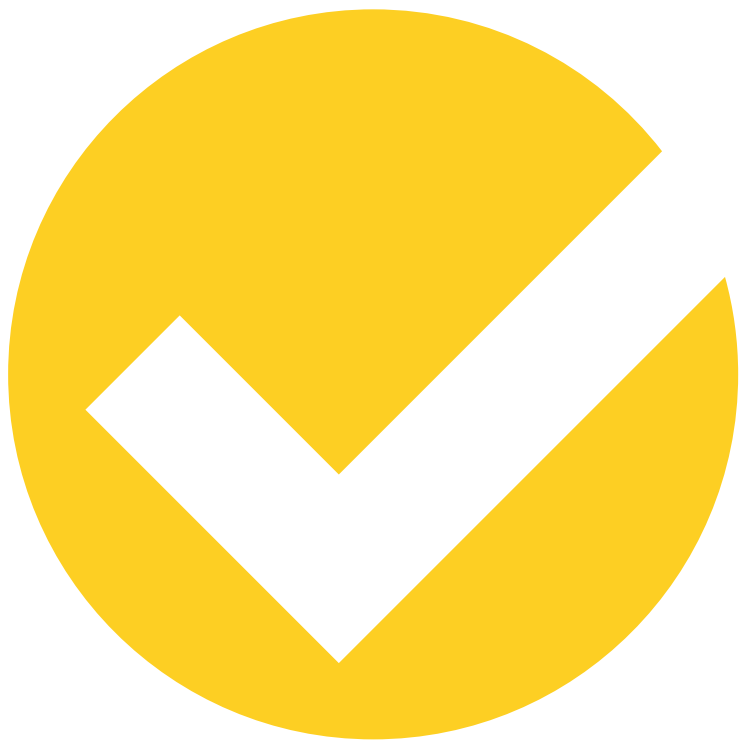

check for  
updates

Supplement: S1 Dataset — (ZIP) [file pone.0264131.s001.zip › sysconf/pubs/collab-patterns/Definitions/logo-updates.pdf]
